# Meta‐analysis suggests negative, but *p*CO_2_‐specific, effects of ocean acidification on the structural and functional properties of crustacean biomaterials

**DOI:** 10.1002/ece3.8922

**Published:** 2022-06-03

**Authors:** Kyle R. Siegel, Muskanjot Kaur, A. Calvin Grigal, Rebecca A. Metzler, Gary H. Dickinson

**Affiliations:** ^1^ 3280 Department of Biology The College of New Jersey Ewing New Jersey USA; ^2^ 3719 Department of Physics and Astronomy Colgate University Hamilton New York USA

**Keywords:** barnacle, biomineralization, calcification, climate change, crab, decapoda, exoskeleton

## Abstract

Crustaceans comprise an ecologically and morphologically diverse taxonomic group. They are typically considered resilient to many environmental perturbations found in marine and coastal environments, due to effective physiological regulation of ions and hemolymph pH, and a robust exoskeleton. Ocean acidification can affect the ability of marine calcifying organisms to build and maintain mineralized tissue and poses a threat for all marine calcifying taxa. Currently, there is no consensus on how ocean acidification will alter the ecologically relevant exoskeletal properties of crustaceans. Here, we present a systematic review and meta‐analysis on the effects of ocean acidification on the crustacean exoskeleton, assessing both exoskeletal ion content (calcium and magnesium) and functional properties (biomechanical resistance and cuticle thickness). Our results suggest that the effect of ocean acidification on crustacean exoskeletal properties varies based upon seawater *p*CO_2_ and species identity, with significant levels of heterogeneity for all analyses. Calcium and magnesium content was significantly lower in animals held at *p*CO_2_ levels of 1500–1999 µatm as compared with those under ambient *p*CO_2_. At lower *p*CO_2_ levels, however, statistically significant relationships between changes in calcium and magnesium content within the same experiment were observed as follows: a negative relationship between calcium and magnesium content at *p*CO_2_ of 500–999 µatm and a positive relationship at 1000–1499 µatm. Exoskeleton biomechanics, such as resistance to deformation (microhardness) and shell strength, also significantly decreased under *p*CO_2_ regimes of 500–999 µatm and 1500–1999 µatm, indicating functional exoskeletal change coincident with decreases in calcification. Overall, these results suggest that the crustacean exoskeleton can be susceptible to ocean acidification at the biomechanical level, potentially predicated by changes in ion content, when exposed to high influxes of CO_2_. Future studies need to accommodate the high variability of crustacean responses to ocean acidification, and ecologically relevant ranges of *p*CO_2_ conditions, when designing experiments with conservation‐level endpoints.

## Graphical abstract

Ocean acidification can affect the ability of marine calcifying organisms to build and maintain mineralized tissue and poses a threat for all marine calcifying taxa, including the physiologically robust crustaceans. Here, we present a systematic review and meta‐analysis on the effects of ocean acidification on the crustacean exoskeleton, assessing both exoskeletal ion content (calcium and magnesium) and functional properties (biomechanical resistance and cuticle thickness). We find negative, but variable, effects of different *p*CO_2_ ranges on exoskeletal properties, suggesting that the crustacean exoskeleton can be susceptible to ocean acidification and warranting further studies.

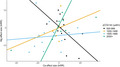

## 1 INTRODUCTION

The phylum Crustacea constitutes a diverse taxonomy of organisms, including species in myriad aquatic environments and ecological roles (Abele, 1974; Bracken et al., 2009; Hultgren et al., 2021). Part of Crustacea's ecological success can be defined by their robust acid‐base regulatory capacities (Leone et al., 2017) and, for some Orders such as *Decapoda* and *Sessilia*, calcified exoskeletons. The mineral‐bearing exoskeletons are a departure from the predominantly chitin‐ and protein‐enforced structures of terrestrial arthropods (Murdock, 2020). Instead, the calcified exoskeletons of crustaceans provide a line of defense from physical insults (deVries et al., 2021) and play a potential role in ionic regulation (Boßelmann et al., 2007). Indeed, Crustaceans are typically able to successfully osmoregulate within the variable physical and chemical conditions found in their marine and coastal environments (Baumann et al., 2014; Whiteley et al., 2018).

Human activities, however, have, can, and will alter the physical and chemical properties of marine environments (Doney et al., 2012). One of the most pressing marine changes in the Anthropocene is the dissolution of carbon dioxide (CO_2_) into marine bodies and subsequent changes in carbonate chemistry (Orr et al., 2005). Reaction of exogenous CO_2_ with seawater produces carbonic acid (H_2_CO_3_), which dissociates into bicarbonate (${\text{HCO}}_{3}^{‐}$) and free hydrogen ions (H^+^). Excess H^+^ interacts with seawater carbonate (${\text{CO}}_{3}^{‐2}$) to further produce more bicarbonate. The excess hydrogen ions gradually decrease ocean pH, providing the basis for the phenomenon of “ocean acidification” (Gattuso et al., 2015; IPCC, 2001; Raven, 2005). Based on current predictions, ocean pH will decrease 0.3–0.5 pH units by the year 2200 (Caldeira & Wickett, 2003; Orr et al., 2005), although changes will vary by regions contingent on the solubility of CO_2_ in local waters, seawater buffering capacity, and ocean mixing patterns (Fabry et al., 2009). H^+^‐mediated depletion of seawater ${\text{CO}}_{3}^{‐2}$ poses a physiological threat to organisms that use carbonate in the production of mineralized tissues, particularly structures composed of calcium carbonate (CaCO_3_). Ocean acidification thus presents a combined insult for organisms that utilize ${\text{CO}}_{3}^{‐2}$: declining supplies of environmental ${\text{CO}}_{3}^{‐2}$ for active mineralization, and potential structural dissolution (Schönberg et al., 2017; Ries et al., 2009).

Mineralization in many calcifying marine invertebrates, such as echinoderms, molluscs, and cnidarians, is directly or indirectly dependent on seawater ${\text{CO}}_{3}^{‐2}$ saturation state (Bednaršek et al., 2020; Cohen et al., 2009; Waldbusser et al., 2015). In contrast, biomineralization in crustaceans has been shown to occur through an extracellular ${\text{HCO}}_{3}^{‐}$‐based mechanism (Cameron, 1985), leaving them less dependent on ${\text{CO}}_{3}^{‐2}$ saturation state. Previous studies on acid‐base maintenance in aquatic crustaceans in high CO_2_ seawater indicate that ${\text{HCO}}_{3}^{‐}$ and calcium (Ca^2+^) regulate hemolymph acid‐base status, at least to some extent, with increases in buffering levels of both ions facilitated partially by exoskeleton dissolution (Defur et al., 1980; Henry et al., 1981; Spicer et al., 2006). The high osmo‐ and iono‐regulatory capacity of crustaceans (Whiteley, 2011), combined with experimental results suggesting positive effects of ocean acidification on their calcification rate (Ries et al., 2009) and growth (McDonald et al., 2009), placed members of the taxon into a category of “less concern” by physiologists regarding the effects of ocean acidification (Ramaglia et al., 2018; Wittman & Portner, 2013). This designation was further buttressed by negligible effects of ocean acidification in multi‐taxa research meta‐analyses (Harvey et al., 2013; Kroeker et al., 2013; Kroker et al., 2010) and physiologically rationalized by some crustaceans’ ability to utilize ${\text{HCO}}_{3}^{‐}$ for exoskeletal mineralization (Cameron, 1989; Cameron & Wood, 1985).

Our current understanding of the physiological responses of marine crustaceans to acidification can be traced to studies of acid‐base regulation under hypercapnic (high CO_2_) conditions. In the latter half of the 20th century, relevant publications focused on crustacean physiological responses following exposure to hypercapnic/hypoxic conditions induced by aerial exposure and, subsequently, hemolymph acidosis (Innes, 1986; Taylor & Whiteley, 1989; Tylor‐Jones & Taylor, 1988). Tyler‐Jones and Taylor (1988) reported efficient pH compensation in hemolymph of the crayfish *Austropotamobius pallipes* following aerial exposure: pH and lactate levels returned to submerged values within 24 h, with noted increases in ${\text{HCO}}_{3}^{‐}$. Similarly, Taylor and Whiteley (1989) observed robust management of hypercapnia‐mediated acidosis in the lobster *Homarus Gammarus*, noting increases in hemolymph concentrations in ${\text{HCO}}_{3}^{‐}$ and Ca^2+^. However, these studies assessed acute responses of decapod crustaceans to hypercapnia, with exposures on the order of days. Of the studies that assessed physiological responses to seawater hypercapnia, a subset modulated seawater carbonate chemistry through *p*CO_2_ (Cameron, 1989; Taylor & Spicer, 1991; Truchot, 1979). Even fewer of these articles reported direct assessments of the effects of *p*CO_2_‐mediated hypercapnia on exoskeletal properties. We located one such publication that recorded positive effects of decreasing pH on carapace Ca^2+^ content in penaeid shrimp. However, the author also observed negative effects of decreasing pH on overall carapace size and molting period, suggesting that the observed increases in Ca^2+^ content were artifacts of a longer intermolt period (Wickens, 1984).

Building upon the mixed effects of high *p*CO_2_ on crustacean calcification observed by Wickens (1984), recent primary research suggests that crustaceans are not universally resistant to the effects of ocean acidification. Nuanced changes in the perception of crustacean's physiological robustness can be derived from growing research interests and concerns on the biological effects of acidification of external seawater, in contrast to earlier work focusing on intra‐organismal hemolymph acidification incurred by aerial exposure. Depending on the selection of ecologically relevant *p*CO_2_ levels and study durations for experiments (McElhany & Busch, 2012), a growing body of the literature describes mixed responses of crustaceans to ocean acidification. For example, exposure of four geographically distinct crab species to worst‐case, but ecologically relevant, acidification levels (ambient pH 8.0; reduced pH: 7.4) produced drastic changes in shell morphology and mineralogy that were slightly abated by a population's natural history in variable environments (Page et al., 2017). Study duration also seemingly influences crustacean responses. Experiments on the order of months to years suggest negative effects of acidification on shell formation in acorn barnacles (Nardone et al., 2018) and survival and exoskeleton properties in cold‐water crabs (Long et al., 2013; Long et al., 2013; Swiney et al., 2016). The complex mineralogy of crustacean exoskeletons, comprised of calcite, Mg‐enriched calcite, amorphous calcium carbonate, and even calcium phosphate in some taxa (Bentov et al., 2016; Bentov et al., 2016; Boßelmann et al., 2007), does not lend the taxon to easy comparisons with other calcifying invertebrates (see Andersson & Mackenzie, 2011 for further elaboration). Therefore, definition of trends in crustaceans’ response to ocean acidification requires rigorous comparison, combination, and analysis of results from potentially disparate studies.

Meta‐analysis provides a framework to quantitatively compare and combine the results from different ecological studies and reveal potential trends in the effects of environmental change (Hedges et al., 1999). Previous multi‐taxa meta‐analyses on ocean acidification's biological effects usually focus on traits relevant to population dynamics and management, such as animal survival, developmental timing, growth rate, and fecundity (Kroeker et al., 2010, 2013). Other meta‐analyses focusing on the biological impacts of ocean acidification center on specific taxa/clades (Bednaršek et al., 2019; Meyer & Riebesell, 2015), biogeographic regions (Hancock et al., 2020; Zunino et al., 2017), or physiological processes in specific taxa (Bednaršek et al., 2021; Chan & Connolly, 2013). To the best of our knowledge, no research synthesis and meta‐analysis has focused solely on crustacean mineralization and exoskeletal properties under ocean acidification, although Bednaršek et al. (2021) strongly informs the need for such an assessment with their meta‐analysis of threshold limits for pH and exposure duration beyond which the physiology and development of decapod crustaceans are negatively affected.

While previous meta‐analyses synthesized various calcification metrics under ocean acidification, “calcification” is usually defined as a subset of organismic growth. “Calcification rate” can be derived from changes in the wet weight of an organism (e.g., quantification of buoyant weight as in Ries et al., 2009) or the size of a mineralized structure (e.g., Jokiel et al., 2008; part of Wood et al., 2008, although this study includes measurements of Ca^2+^ content as well). While these measurements are important to understand organism‐level effects of ocean acidification on crustaceans, they do not address changes in crustacean exoskeletal composition and function under ocean acidification that could have important physiological and ecological consequences. Here, we present results from a systematic review and meta‐analysis on crustacean mineralization under ocean acidification in the structural and functional sense, attempting to answer three related questions: (1) Does ocean acidification (high seawater *p*CO_2_) alter the ionic composition of the crustacean exoskeleton in a predictable way?; (2) Does ocean acidification lead to functional changes of the exoskeleton, that is, in biomechanical properties (microhardness and shell strength) and cuticle thickness, that could have ecologically relevant effects?; (3) Given the variability in crustacean responses to ocean acidification, are there any biological (taxonomic order, life history stage, biogeographic region, exoskeletal region of sample) factors that affect crustacean biomineralization under ocean acidification?

## 2 METHODS

### 2.1 Systematic review

Initial searches were conducted in English on multiple electronic databases using combinations of Boolean phrases (itemized in the following paragraph). Databases in the search included: SCOPUS, Biological & Agricultural Index Plus (H.W. Wilson), Biological Sciences Database, EBSCOhost Academic Premier, General Science FullText, and Applied Science & Technology Full Text. Searches were conducted during May and June 2020 on references available before, and including, 30 June 2020. Two exceptions were made for datasets collected in the Dickinson laboratory at The College of New Jersey. At the time, one was in the manuscript preparation phase (Dickinson et al., 2021, focused on the Tanner crab *Chionoectes bairdes*). The other is an unpublished dataset from experiments using the acorn barnacle *Amphibalanus amphitrite*.

Search terms comprised permutations of three main factors: environmental treatment (ocean acidification OR pH OR *CO2), organism of interest (by species/common name: crustacea* OR crab* OR barnacle* OR krill* OR lobster* OR shrimp* OR copepod* OR isopod* OR amphipod*; by order: crustacea** OR branchiopod* OR isopod* OR amphipod* OR decapod*), and trait measured (***minerali*ation OR calcification OR shell OR growth OR physiology OR development OR life history). Search parameters including species and orders were necessary as article titles and abstracts tended to favor organism description at either the order‐ or species‐level, but not both (authors’ personal observations). While primary research on copepods and branchiopods was not included in the data extraction and analysis, the terms were used to account for comparative studies on multiple crustacean species. Wildcard symbols (i.e., *) were used to account for plural terms (i.e., both “crab” and “crabs”), multiple terms with the same root (i.e., “crustacea**” for “crustacea,” “crustacean,” and “crustaceans”), and regional differences in spelling (i.e., ‘minerali*ation’ for both “minerali*z*ation” and “minerali*s*ation”).

In addition to primary database searches, the “References” section of articles deemed acceptable for full‐text review were searched for relevant articles. Ocean acidification‐specific articles were also queried on the papers database from Pangaea (https://www.pangaea.de/). Google Scholar profiles of researchers known to publish on crustaceans and ocean acidification (Table S1) were also searched.

Articles passed preliminary inclusion criteria if details available in the title and abstract suggested a focus on the effects of ocean acidification on the physiology of crustaceans. Although our primary focus was on structural and functional changes in the crustacean exoskeleton under ocean acidification, we searched with a broad suite of physiological parameters due to variability in term use between publications. For example, the title for Long, Swiney, Harris, et al. (2013) uses “calcification” to refer to measurements of carapace mineralogy in *Paralithodes camtschaticus* and *Chionoecetes bairdi*. However, “calcification” can also refer to measurements of exoskeletal growth and dissolution over time, such as in Ries et al. (2009). Here, “physiology” included, but was not limited to, measurements for: calcification, respiration, heart rate, metabolism, organismic growth, shell properties [growth, hardness, thickness, and mineralogy], fecundity, and/or mortality. Articles on terrestrial and freshwater crustaceans were excluded from the analysis but included in the overall search results. Similarly, articles that measured molecular changes (i.e., transcriptomic and proteomic studies) and/or physiological changes not focused on exoskeletal properties (i.e., respiration, heart rate, metabolism, fecundity, and mortality) were excluded from analysis but included in the overall search results. All articles that passed the first inclusion criteria were collated into an Excel database (see Supplemental Materials for file) to determine the total number of unique articles located.

Articles then had to pass a second set of inclusion criteria prior to data extraction. Criteria for the second round included as follows: (1) access to parameter mean values with a metric of variance (standard error, standard deviation, 95% confidence intervals, or interquartile ranges) from article tables, figures, text, or supplemental materials; (2) changes in seawater pH using manipulation of *p*CO_2_ (i.e., studies that altered pH using direct acid addition were not included (Cornwall & Hurd, 2016); (3) use of a “control” group for studies that modulated food availability and/or multiple seawater parameters (i.e., a group that received standard operating procedure levels of food, or were exposed to ambient temperature/salinity but increased *p*CO_2_). In addition, as copepods do not produce mineralized exoskeletons (Kaneko & Colwell, 1975), we removed any articles that assayed only copepod responses to ocean acidification at this point in the review.

### 2.2 Data inspection and extraction

Data points were recorded from an article that passed the second inclusion criteria if the paper: (1) presented quantitative data for high *p*CO_2_‐ and ambient‐exposed groups, (2) structured its experiments on separate populations (technical replicates, such as multiple panels within the same aquarium, would only be counted as a single data point), and (3) reported mean and variance values at the individual animal level (i.e., not as summary values for a colony, tank, beaker). Multiple data points could therefore be extracted from a single article. We then surveyed the methods sections and figures to qualify which physiological parameters were assayed in the article and, if those parameters measured exoskeletal composition and function, their associated methodologies. For composition, we focused on studies that quantified mineral ion levels of exoskeletal samples. Measurement techniques included ion chromatography, ICP‐MS, ICP‐OES, AAS, and EDS (Table A1). For function, we included measurements of biomechanical properties such as microhardness and shell strength, as well as total cuticle thickness (Table A2). At this point in the systematic review, we noticed that most publications on crustacean exoskeleton research used species from the Orders *Decapoda* and *Sessilia*. We decided to focus our analysis on decapods and *Sessilia* barnacles based on the larger sample sizes their literature afforded.

Where enumerated, average values and measures of variation (standard deviation, standard error of the mean, confidence intervals, and interquartile ranges) were obtained directly from article text and/or tables. If values were not readily available from main or supplementary texts and/or tables, mean and variance values were calculated from article figures using WebPlotDigitizer (Rohatgi, 2020), downloaded to individual CSV files, and added to a parameter‐specific Excel file. All extraction files can be found in the Supplemental Materials. If average and variance values could not be confidently obtained by either method, the corresponding author of the paper was contacted and raw data requested. For studies that included multiple decapod or barnacle species, each species was included as a separate data point. If measurements were taken at multiple time points during the study, we used values from the last time point for analysis and subgroup categorization. If multiple structures were assayed (i.e., the carapace and chelae of a crab), all comparisons were included. Structural and biomechanical properties vary between calcified regions in crustaceans, usually concomitant with different ecological functions (Boßelmann et al., 2007; Chen et al., 2008). We therefore included all relevant data points regardless of anatomical location to build a holistic model of crustacean exoskeletal structure and function under ocean acidification.

As meta‐analytic models use standard deviation for computations, variances presented in other formats were converted to standard deviation: Conversion equations can be found in Document S1. Sample sizes were obtained from the article and/or supplemental materials as available. If sample sizes could not be obtained directly, we estimated the values by extracting survival rates from figures or text, as available, and calculating survivorship numbers based on the initial sample size (as presented in figures or text). For ionic content analysis, datapoints expressed as percentages or concentration per unit mass, mean values and standard deviations were standardized to percentages of tissue dry mass (used for 47 of 97 data points) or μmol/mg (used for 12 of 97 data points), prior to log transformation, to minimize heterogenous effects produced by differences in scale. Equations for the latter conversion can be found in the Document S2. The remaining 38 data points, where ionic content analysis datapoints were not expressed as percentages or concentration per unit mass, remained in their specialized units prior to log transformation.

Ambient pH and *p*CO_2_ levels were assumed to reflect average levels at an organisms’ collection site. For multi‐generational studies, data were included if non‐transgenerational control and OA groups were included and met the preceding criteria. Seawater *p*CO_2_ values were obtained from the text or supplemental materials where available. If a paper did not calculate *p*CO_2_, values were obtained using the “seacarb” package in R (v. 3.2.16; Gattuso et al., 2020) to derive *p*CO_2_ from pH, dissolved inorganic carbon (DIC), and total alkalinity (TA) values.

For biological predictors of crustacean responses, the “order” co‐variate was determined by species entry in the World Register of Marine Species (WoRMS; https://marinespecies.org). Ultimately, we decided to focus on the orders *Decapoda* and *Sessilia*, as they constituted the majority of crustacean species used for studies assessing the effects of ocean acidification on exoskeleton‐related parameters. Geographic zones for “biogeography” were determined based on collection site latitudes included in articles’ Methods sections. Zones for “Biogeography” were divided between tropical (roughly, 0°–35° N/S), temperate (35°–50° N/S), and polar (50° + N/S) (American Meteorological Society, 2000). Life history stages were determined based on in‐text specification.

### 2.3 Meta‐analysis

In accordance with previous meta‐analyses on organismal responses to ocean acidification (Cattano et al., 2018; Kroeker et al., 2010, 2013; Wittmann & Portner, 2013), comparisons for this meta‐analysis used the log‐transformed response ratio common to ecological research syntheses (see Hedges et al., 1999). The log‐response ratio is well‐suited for ecological syntheses in that it computes a unitless value for direct comparison on the effects of the experimental treatment. We calculated the response ratio (*L*) for each data point using the following equation:

$$L=\ln\left(\mathrm{RR}\right)=\ln\left(\frac{X_{E}}{X_{C}}\right)=\ln\left(X_{E}\right)‐\text{ln}\left(X_{C}\right)$$

Where X is the mean value for the biomineralization response under acidified (subscript E) or ambient (subscript C) conditions. The variance for each response ratio was determined using the following equation (a la Hedges et al., 1999):

$$S_{\mathrm{pooled}}=\frac{{\left(S_{E}\right)}^{2}}{n_{E}X_{E}^{2}}+\frac{{\left(S_{C}\right)}^{2}}{n_{C}X_{C}^{2}}$$

where *S* and *n* are the standard deviation and sample sizes for the respective group denoted in the subscript.

Analyses and visualizations were conducted in R (version 3.6.2). All code, Excel, and CSV files used for this article are available on Dryad (URL available in the Data Availability Statement). All analyses were run using the R package “metafor” (v2.4–0; see Viechtbauer, 2010). Random‐effects models were used to account for both within‐study (S_pooled_) and between‐study (τ^2^) heterogeneity. We used the DerSimonian‐Laird method (“DL” in metafor) to approximate τ^2^. Statistical significance was determined by the 95% confidence intervals for a summary response ratio: the effect size (the magnitude of the response ratio) was considered significant (*α* = .05) if the confidence intervals did not overlap with 0.

As ocean acidification in natural systems results from influxes of CO_2_, we decided to group effect sizes by *p*CO_2_ levels of the study's acidified treatment. These bins corresponded to acidified ranges of 500–999, 1000–1499, 1500–1999, and 2000+ µatm, in line with groupings previously used by other meta‐analyses (Hancock et al., 2020; Wittmann & Portner, 2013). All analyses were run on these groupings or, if broken down into further subgroups (i.e., by element, animal developmental stage, biogeographic region, and taxonomic order), re‐analyzed on the new subset of effect sizes. Residual heterogeneity of the models was assessed through the *Q_E_* value, a metric of how well the model explains variance for the summary effect size. A large, statistically significant *Q_E_* value indicates substantial heterogeneity not explained by the model and/or subgroup, suggesting other explanatory factors are necessary to model a biological response more accurately.

Simultaneous changes in Ca^2+^ and Mg^2+^ levels were analyzed by applying a general linear regression using the R function “lm.” Ca^2+^ and Mg^2+^ effect sizes included in the model were weighted by both *S_pooled_* and τ^2^.

## 3 RESULTS

### 3.1 Systematic review

**FIGURE 1:**
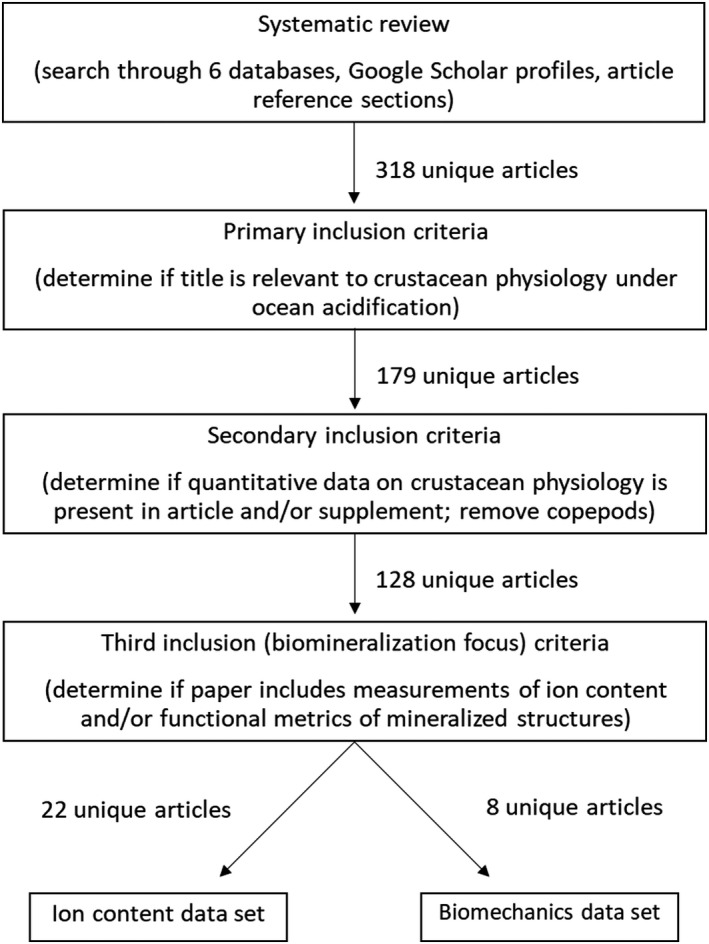
Flow through chart of systematic review and exclusion criteria. From 318 unique articles on ocean acidification's impact on crustacean physiology, we located <30 articles that assayed changes in structural and biomechanical properties in the exoskeletons of crustacean animals (Orders: *Decapoda* and *Sessilia*) under ocean acidification

Combined searches across the 6 databases, reference lists, and ocean acidification‐specific authors list returned 318 unique articles that met the first inclusion criteria (Figure 1). One article (Wei et al., 2014, *Acta Ecologica Sinica*) was excluded as the full text in English could not be obtained. Generally, searches on SCOPUS returned 10 to 20 times more potential hits than any other search platform (Figure A1). This was followed by Science Direct, albeit an order of magnitude lower than SCOPUS. Both SCOPUS and Science Direct are services of Elsevier, whose journals in a 2015 estimate publish roughly 20% of all articles in the natural and medical sciences (Larivière et al., 2015). In addition, SCOPUS utilizes a Boolean‐based search algorithm, known to perform better in search tasks than hypertext‐based platforms (Wolfram & Dimitroff, 1998).

In total, 179 unique articles from the initial 318 met the first inclusion criteria, where the title and abstract suggested that general properties of crustacean physiology (including biomineralization/calcification) under ocean acidification were assayed. Removal of articles on copepods to focus on crustaceans that produced mineralized exoskeletons reduced the article count to 128 unique entries.

### 3.2 Ion concentration

The final ion concentration dataset included 22 unique articles (Table A1), totaling 96 data points/effect sizes. Measurements of Ca^2+^ and Mg^2+^ were reported in roughly equal measure: 51 data points vs 45 data points, respectively. One article included measurements of strontium (Page et al., 2017). However, we decided to focus on Ca^2+^ and Mg^2+^ concentrations given their frequency of measurement in the studies identified by our systematic review. We initially identified Wickens (1984) as a potential article to include in our dataset, as the experiments used CO_2_ to decrease seawater pH and measured exoskeletal Ca^2+^ content in penaeid shrimp. However, neither *p*CO_2_ values nor carbonate chemistry parameters are included and, given the age of the article, acquisition of those values through personal communications with the author was not possible.

### 3.3 Biomechanical properties and cuticle thickness

The biomechanics dataset included fewer papers than the ion concentration dataset. In its final iteration, 8 unique articles were included, with 37 data points (Table A2). This comprised 19 data points from 6 papers for hardness (exoskeleton resistance to physical deformation, including measurements of microhardness and shell strength), and 18 data points from 6 articles for exoskeleton thickness (including only whole cuticle measurements). While one article described the effects of ocean acidification on exoskeleton biophotonics (Taylor et al., 2015), we did not pursue this characteristic due to the limited sample size.

### 3.4 Meta‐analysis: ionic concentrations

**FIGURE 2:**
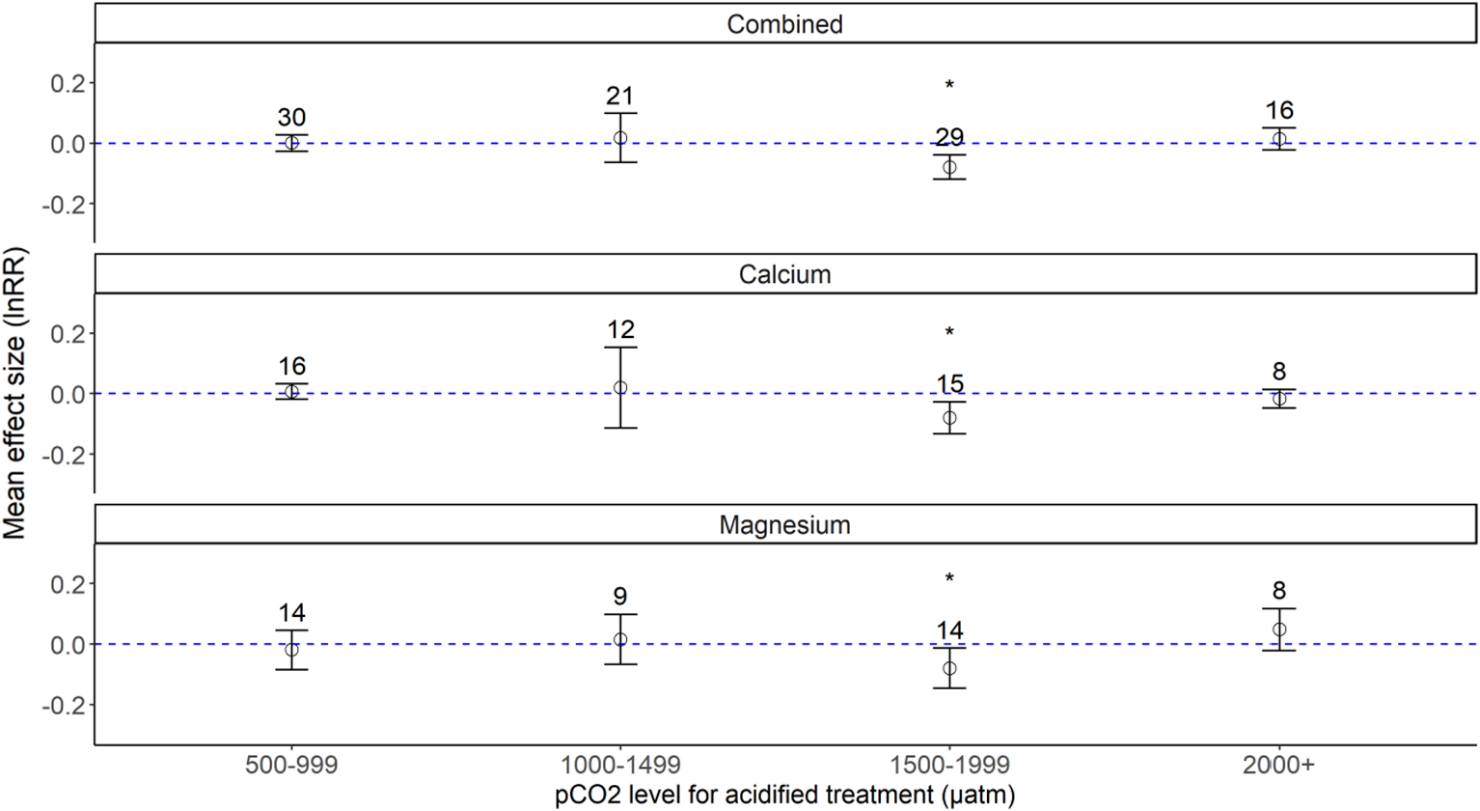
Meta‐analysis of Ca^2+^ and Mg^2+^ content in the crustacean exoskeleton under increasing seawater *p*CO_2_. Figures present the mean (open dot) and 95% confidence intervals for Ca^2+^ and Mg^2+^ combined (a), Ca‐only (b), and Mg‐only (c) summary effect sizes. Bins are *p*CO_2_ levels for high CO_2_ treatments. Numbers directly above the brackets indicate the number of data points for each bin. Effect sizes that are statistically different than 0 (with *p* < .05) are marked with an asterisk

Overall, element concentration (combined Ca^2+^ and Mg^2+^) effect sizes under ocean acidification, regardless of any biological or experimental designation, tended to be comparable with ambient levels in three of the *p*CO_2_ bins, 500–999, 1000–1499 and 2000+ µatm. None of these effect sizes indicated a statistically significant difference between exoskeleton composition from animals exposed to ambient versus high CO_2_ conditions. In contrast, for the 1500–1999 µatm *p*CO_2_ bin, combined ion levels showed a significant decrease as compared with ambient conditions (Figure 2a). Large residual heterogeneity was noted for all *p*CO_2_ bins, with a *Q* range from 46.2 to 91.5 across bins (Table A3). While the 1500–1999 µatm bin displayed a clear decrease in exoskeleton ion content under high CO_2_ conditions, the variance in individual effect sizes, and the variance for all responses in the dataset, were not explained by CO_2_ level alone.

As mineralization responses of crustaceans to environmental stress, particularly ocean acidification, are not uniform and can vary between species and elements quantified (for differing responses, see Long, Swiney, Harris, et al., 2013; Page et al., 2017; Small et al., 2015, as examples), we also analyzed the changes in Ca^2+^ and Mg^2+^ levels separately under high *p*CO_2_ conditions. Effect sizes for Ca^2+^ levels showed a tendency to increase under elevated CO_2_ for the 500–999 µatm and 1000–1499 µatm bins. A statistically significant decrease from 0 for the 1500–1999 µatm bin was observed, indicating Ca^2+^ levels in samples from OA‐exposed animals were lower than ambient‐exposed animals across the included studies (Figure 2b). Ca^2+^ tended to decrease under high CO_2_ in the 2000+ µatm range, but the effect size was not significantly different from 0. In addition, residual heterogeneity for the 500–999, 1000–1499, and 1500–1999 µatm bins was large and statistically significant (*Q* range: 30.42–73.41; Table A4), indicating that the summary effect size is statistically different than the individual effect sizes or that changes in Ca^2+^ levels varied considerably between studies. However, the *Q* value for the 2000+ µatm summary effect size was not statistically significant (11.75, *p* = .10, df = 7). While a non‐significant *Q* value could suggest that Ca^2+^ effect sizes in the 2000+ µatm range are more homogenous with lower between‐study variance, the *Q* statistic is known to be less responsive to the presence of heterogeneity at lower sample sizes (Huedo‐Medina et al., 2006) and, therefore, not entirely indicative of consistent effect sizes.

The effect sizes for Mg^2+^ suggested a slightly different response than calcium (Figure 2c). Mg^2+^ effect sizes in the 500–999 µatm range tended to be negative (*p* = .5521), unlike the positive effect sizes noted for Ca in the lowest *p*CO_2_ bin. Mg^2+^ levels tended to increase under high CO_2_ in the 1000–1499 µatm range (*p* = .7204). Like Ca^2+^ summary effect sizes, Mg^2+^ effect sizes were significantly reduced in high CO_2_ conditions within the 1500–1999 µatm regime (*p* = .0178). Mg^2+^ levels tended to increase in acidified conditions for the 2000+ µatm bin (*p* = .1731), juxtaposed to the tendency to decrease for Ca^2+^ effect sizes in this *p*CO_2_ range. However, large residual heterogeneity was noted for all Mg^2+^ summary effect sizes regardless of *p*CO_2_ regime, as evidenced by large, statistically significant *Q* statistics (Table A5). Thus, individual effect sizes for Mg^2+^ content under high CO_2_ greatly varied in terms of direction and magnitude, suggesting that *p*CO_2_ conditions alone are not sufficient to predict changes in Mg content in crustacean exoskeleton under high CO_2_ conditions.

**FIGURE 3:**
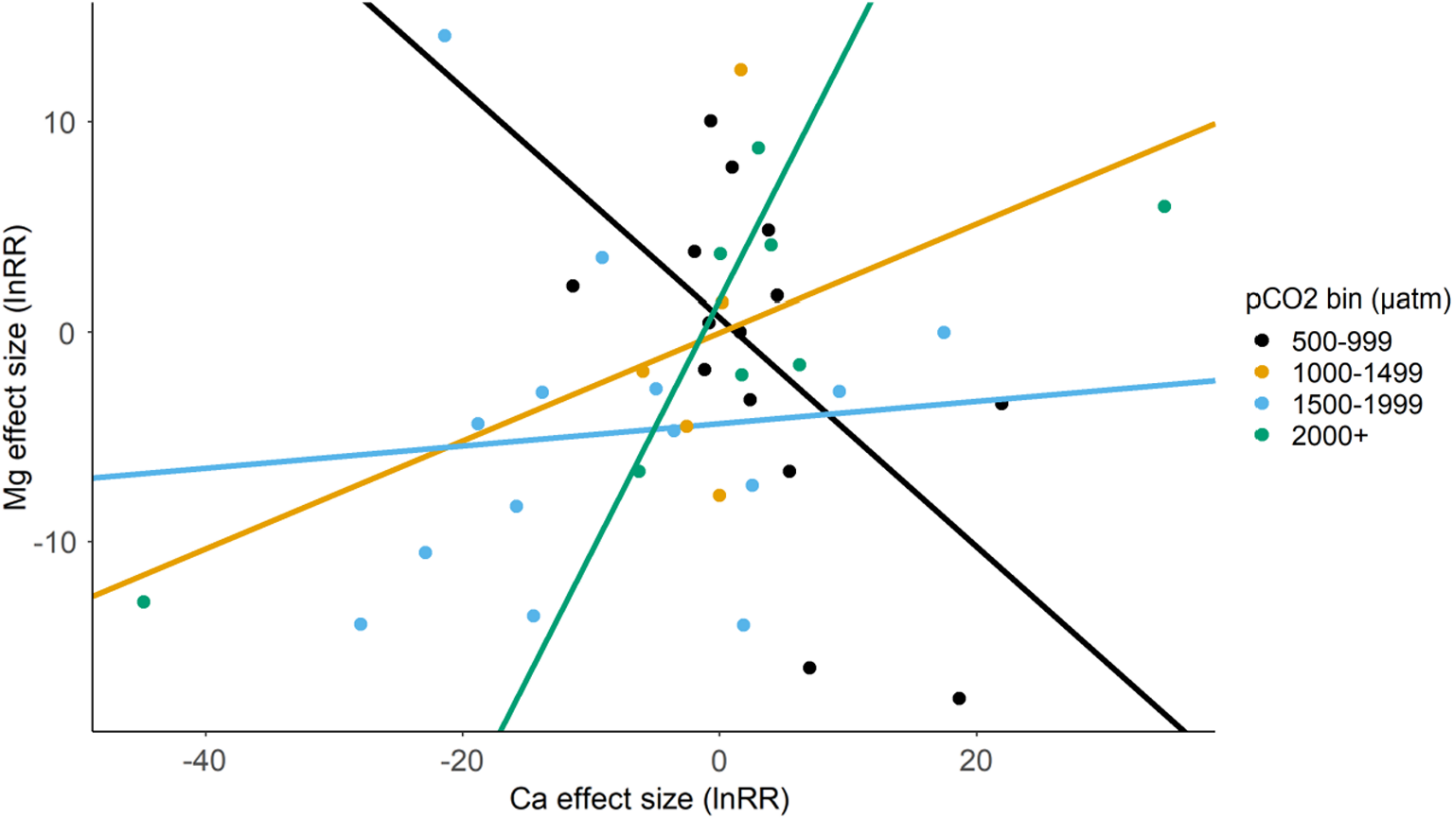
Linear regression of Mg^2+^ effect size by Ca^2+^ effect size from the same experiment suggests differing calcification responses to ocean acidification under increasing *p*CO_2_ regimes

In a previous study, mineralized tissue from 7 body regions of the lobster *Homarus americanus* raised in ambient seawater were found to vary in Ca^2+^ and Mg^2+^ levels, but Ca:Mg ratios were consistent among body regions (Mergelsberg et al., 2019). Similarly, the Ca:Mg ratio was maintained under high experimental acidification (ambient: 800 µatm; OA: 8000 µatm) in the carapace of juvenile crabs, *C*. *sapidus*, collected from a comparable geographic and climatic region (Glandon et al., 2018), suggesting that stability of the Ca:Mg ratio is physiologically sustained even under acidified conditions. To determine how ocean acidification affects the Ca:Mg ratio in the crustacean exoskeleton, we applied a linear regression to model the Mg^2+^ effect size as a product of the Ca^2+^ effect size for Ca^2+^ and Mg^2+^ data acquired from the same experiment (Figure 3). A total of 41 unique effect sizes from 18 manuscripts were included.

For response ratios within the 500–999 µatm range (*n* = 14), Ca^2+^ and Mg^2+^ displayed a significant, negative relationship (coefficient: −0.5462, *p* = .0301) with a noticeably strong explanatory correlation of changes in Ca^2+^ for changes in Mg^2+^ (adjusted *R* ^2^: .2765). The coefficient of the linear model for the 500–999 µatm range coincided with the summary effect sizes for Ca^2+^ and Mg^2+^ noted in Figure 2b and c (Ca^2+^ tended to increase while Mg^2+^ tended to decrease; their negative relationship was statistically significant). In the 1000–1499 µatm range (*n* = 8), Ca^2+^ and Mg^2+^ had a significant, positive relationship (coefficient = 0.2579, *p* = .0214) with a large explanatory correlation (adjusted *R* ^2^ = .5498). Again, the linear model and the summary effect sizes for calcium and magnesium in Figure 2b and c matched in terms of directionality (Ca^2+^ and Mg^2+^ tended to increase, with their positive relationship being significant). Linear models for the Mg‐by‐Ca response ratios from the 1500–1999 µatm range (*n* = 8) and the 2000+ µatm range (*n* = 5) did not exhibit statistically significant directionality (1500–1999 µatm: 0.0531; 2000+ µatm: 1.206). The explanatory power, as evidenced by the adjusted *R* ^2^ values, of these models was negligible (1500–1999 µatm adjusted *R* ^2^: −.0743; 2000+ µatm adjusted *R* ^2^: −.0445).

Given the significant decreases in both Ca^2+^ and Mg^2+^ levels noted in the 1500–1999 µatm regime, we were surprised that the two elements did not significantly co‐vary in our regression analysis. Manual inspection of the effect size comparisons included in the 1500–1999 µatm range indicated that 8 of the 14 total comparisons showed Ca^2+^ and Mg^2+^ levels co‐varying in the same direction. Of these eight, seven comparisons pulled from Page et al. (2017), and 1 value from Swiney et al. (2016), showed statistically significant covariance in the same direction between Ca^2+^ and Mg^2+^ levels with moderately high explanatory value (0.4129; adjusted *R* ^2^: .4888; *p* = .0485). The remaining 6 comparisons, pulled from Coffey et al. (2017), Nardone et al. (2018), and the unpublished *A*. *amphitrite* dataset, did not show significant covariance between Ca^2+^ and Mg^2+^ levels under CO_2_ influxes (−0.4289; adjusted *R* ^2^ = .2201; *p* = .1954).

As noted above (Figure 2), Ca^2+^ and Mg^2+^ effect sizes were significantly lower for animals within the 1500–1999 µatm range as compared with those at ambient *p*CO_2_. However, Ca^2+^ and Mg^2+^ levels did not significantly decrease in the highest *p*CO_2_ range (2000+ µatm), nor did effect sizes differ significantly from ambient levels. Indeed, the large residual heterogeneity suggested by statistically significant *Q* values (Tables A4 and A5) suggested that other factors beyond the Ca^2+^:Mg^2+^ relationship could influence Ca^2+^ and Mg^2+^ levels in crustacean exoskeletons under ocean acidification/high CO_2_. To better contextualize these trends, we examined biological (taxonomic order, biogeographic region, life history stage, and anatomical region) distinctions between each of our *p*CO_2_ bins.

We analyzed each *p*CO_2_ bin by one of four categorical subgroupings: taxonomic order, biogeographical region, life history stage, and anatomical region of sample. For Ca^2+^, the statistically significant decrease under acidification of 1500–1999 µatm was seemingly driven by adult decapods from temperate collection regions (Table 1). When we investigated the articles included in the 1500–1999 µatm set, we noted that data from Page et al. (2017) contributed consistently negative effect sizes accrued from adult decapod animals. Removing all 7 data points collected from Page et al. (2017) and re‐analyzing the dataset (a sensitivity test) returned a negative, but not statistically significant, summary effect size (lnRR = −0.0344; *p* = .2931). No specific biological factors significantly influenced the summary effect size in the 2000+ µatm range: only the subgroup of “Adult” animals returned an effect size statistically different from 0. *Q* values for each subgroup were large and statistically significant, indicating high variance in Ca^2+^ effect sizes between experiments or, depending upon the subgroup, small sample sizes (Tables S3–S5).

**TABLE 1:** Subgroup analysis of exoskeletal Ca^2+^ levels under high seawater *p*CO_2_ suggests mixed responses in crustaceans. Values are the log‐transformed summary effect size. n = number of data points included in each subgroup

	pCO_2_ bin			
	500–999	1000–1499	1500–1999	2000+
Calcium				
Overall	0.0065 (*n* = 16)	0.0193 (*n* = 12)	−0.0796^*^ (*n* **= 15**)	−0.017 (*n* = 8)
Taxonomy				
*Decapoda*	0.032 (*n* = 11)	0.0465 (*n* = 9)	−0.0992^*^ (*n* = 13)	−0.0175 (*n* = 7)
*Sessilia*	0.0130 (*n* = 5)	−0.0616 (*n* = 3)	0.0109 (*n* = 2)	−0.0225 (*n* = 1)
Biogeography				
Polar	0.0034 (*n* = 10)	0.0645 (*n* = 1)	−0.0547 (*n* = 6)	−0.0225 (*n* = 1)
Temperate	0.0132 (*n* = 5)	−0.0863 (*n* = 7)	−0.1070^*^ (*n* = 9)	−0.0175 (*n* = 7)
Tropical	0.0082 (*n* = 1)	0.1651 (*n* = 4)	ND	ND
Life history stage				
Larvae	ND	−0.1828 (*n* = 2)	ND	ND
Juvenile	−0.0107 (*n* = 6)	−0.0135 (*n* = 7)	−0.0348 (*n* = 3)	0.0153 (*n* = 2)
Adult	0.0199 (*n* = 10)	0.2999^*^ (*n* = 3)	−0.0964^*^ (*n* = 12)	−0.0366^*^ (*n* = 6)
Anatomy				
Carapace	0.0130 (*n* = 6)	0.0704 (*n* = 5)	−0.1298^*^ (*n* = 7)	−0.0184 (*n* = 3)
Chelae	0.0277 (*n* = 3)	0.0743 (*n* = 3)	−0.0704 (*n* = 5)	−0.0167 (*n* = 3)
Base plate	0.0011 (*n* = 2)	ND	0.0093 (*n* = 1)	ND
Parietal plate	0.0151 (*n* = 2)	ND	0.0123 (*n* = 1)	ND

ND, no data available for subgroup.

* *p* < .05.

Subgroup analysis within *p*CO_2_ bins for the Mg^2+^ dataset did not point to any consistent biological factors that drove the results, although the subgroups for temperate animals within the 1500–1999 µatm set and “chelae” within the 2000+ µatm set returned effect sizes significantly different than 0 (Table 2). However, as Page et al. (2017) reported values for both Ca^2+^ and Mg^2+^ concentrations, we performed a similar sensitivity assessment as for the Ca^2+^ 1500–1999 µatm grouping. Re‐analysis following removal of Mg^2+^ effect sizes accrued from Page et al. (2017) in the 1500–1999 µatm range returned a negative, but not statistically significant, summary effect size (lnRR = −0.0301; *p* = .6080). Similar to subgroups of Ca^2+^ effect sizes, *Q* values for Mg^2+^ summary effect sizes grouped by biological co‐variates were also large and statistically significant, although this could be biased due to small sample sizes for certain subgroups (Tables A9–A13).

**TABLE 2:** Subgroup analysis of exoskeletal Mg^2+^ levels under high seawater *p*CO_2_ suggests mixed responses in crustaceans. Values are the log‐transformed summary effect size. *n* = number of data points included in each subgroup

	pCO_2_ bin			
	500–999	1000–1499	1500–1999	2000+
Magnesium				
Overall	−0.0196 (*n* = 14)	0.0152 (*n* = 9)	−0.0799* (*n* = 14)	0.0483 (*n* = 8)
Taxonomy				
*Decapoda*	−0.005 (*n* = 9)	−0.0657 (*n* = 8)	−0.0657 (*n* = 12)	0.0687 (*n* = 7)
*Sessilia*	−0.0391 (*n* = 5)	−0.081 (*n* = 1)	−0.1479 (*n* = 2)	−0.101 (*n* = 1)
Biogeography				
Polar	−0.0093 (*n* = 8)	0.0407 (*n* = 2)	0.0432 (n = 5)	0.0267 (n = 2)
Temperate	−0.0367 (*n* = 5)	−0.0307 (n = 5)	−0.1299* (n = 9)	0.0541 (n = 6)
Tropical	0 (*n* = 1)	0.1391 (n = 2)	ND	ND
Life history stage				
Larvae	ND	−0.0275 (*n* = 2)	ND	ND
Juvenile	0.0066 (*n* = 4)	0.0318 (*n* = 4)	−0.1479 (*n* = 2)	0.0267 (*n* = 2)
Adult	−0.0315 (*n* = 10)	0.0484 (*n* = 3)	−0.0657 (*n* = 12)	0.0541 (*n* = 6)
Anatomy				
Carapace	0.0151 (*n* = 6)	0.0338 (*n* = 6)	−0.058 (*n* = 7)	0.0212 (*n* = 3)
Chelae	−0.0826 (*n* = 3)	0.3272 (*n* = 1)	−0.0771 (*n* = 5)	0.1351^*^ (*n* = 3)
Base plate	0.0043 (*n* = 2)	ND	−0.1768 (*n* = 1)	ND
Parietal plate	0.0083 (*n* = 2)	ND	−0.1123 (*n* = 1)	ND

ND, no data available for subgroup.

* *p* < .05.

### 3.5 Meta‐analysis: mechanical properties and cuticle thickness

Exposure to elevated *p*CO_2_ has the potential to alter functional properties of the exoskeleton. As such, we assessed how elevated CO_2_ altered exoskeleton biomechanical properties and thickness. From 8 distinct papers (Table A2), we extracted 37 unique data points for the biomechanical properties (resistance to mechanical insult and breakage, such as microhardness and shell strength; *n* = 19 data points) and total cuticle thickness (*n* = 18 data points).

**FIGURE 4:**
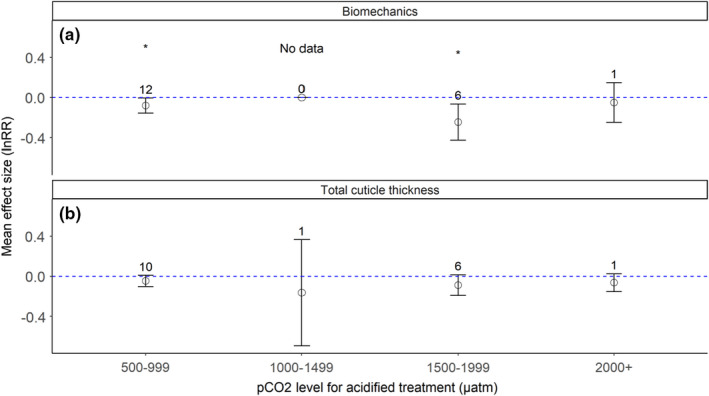
Meta‐analysis of biomechanical properties and exoskeletal thickness of the crustacean exoskeleton. Figures present the mean (open dot) and 95% confidence intervals for exoskeleton (a) mechanical properties (microhardness, shell strength) and (b) total cuticle thickness. Bins are *p*CO_2_ levels for high *p*CO_2_ treatments. Numbers directly above the brackets indicate the number of data points for each bin. Effect sizes that are statistically different from 0 (*p* < .05) are marked with an asterisk

Biomechanical properties (microhardness or shell strength) showed negative summary effect sizes for all three *p*CO_2_ bins where data were available (Figure 4a). Effect sizes were significantly lower than 0 for the 500–999 µatm (*p* = .0380) and 1500–1999 µatm (*p* = .0078) ranges. However, large residual heterogeneity was noted for both bins, with statistically significant *Q* values (Table A6). Our systematic review did not identify any publications that measured exoskeleton hardness within a 1000–1499 µatm *p*CO_2_ range for the acidified treatment. The limited sample size of 1 data point for the 2000+ µatm bin seemed to continue the trend of decreased exoskeleton hardness under acidification but is based on one effect size.

Cuticle thickness tended to decrease under high CO_2_ conditions across *p*CO_2_ bins (Figure 4b), but no summary effect sizes were significant as compared with ambient conditions. Single data points comprised the 1000–1499 µatm and 2000+ µatm effect sizes, with negative effect sizes for both. Both the 500–999 µatm and 1500–1999 µatm summary effect sizes for thickness did not have statistically significant heterogeneity (Table A7).

## 4 DISCUSSION

Crustaceans encompass a diverse and cosmopolitan group of organisms which are often considered to be less susceptible to ocean acidification than other taxonomic groups (Harvey et al., 2013; Kroker et al., 2010; Kroeker et al., 2013; Whitely, 2011). The success of crustaceans is largely attributed to their robust physiological regulation, in part driven by physical and chemical use of their calcified exoskeletons (deVries et al., 2021; Whiteley, 2011). Here, we present the results from a systematic review and meta‐analysis on the effects of elevated *p*CO_2_ seawater on structural and functional properties of the crustacean exoskeleton. We aimed to assess if ocean acidification altered crustacean exoskeletal properties in a predictable way and, potentially, if such changes could have ecologically relevant effects. From searches across 6 databases, 318 article reference sections, Google Scholar profiles of known researchers in the field, and related ocean acidification websites, we ultimately located less than 30 total articles to include for our ion content and functional property datasets (Document S3). Exposure to moderately high levels of elevated *p*CO_2_ seawater (1500–1999 µatm) resulted in a statistically significant reduction in exoskeletal Ca^2+^ content, Mg^2+^ content, and hardness, as compared with animals held under ambient *p*CO_2_ conditions. Exposure to end‐of‐century *p*CO_2_ levels (500–999 µatm) also significantly decreased biomechanical properties. High levels of heterogeneity, as evidenced by statistically significant *Q_E_* values, were noted for most analyses. However, the relationship between Ca^2+^ and Mg^2+^ content under low to moderate *p*CO_2_ levels (<1500 µatm) significantly covaried in predictable ways.

### 4.1 Systematic review

It is difficult to compare the quality of our systematic review's search results (318 articles following the first inclusion criteria, down to ~30 articles included in the meta‐analyses) to other published systematic reviews on the biological effects of ocean acidification. Many meta‐analyses do not include the number of articles identified, but ultimately excluded, in their report. However, we can approximate that the effects of ocean acidification on crustaceans is understudied compared with other marine calcifiers. For example, Meyer and Riebesell (2015) located 19 unique articles on calcification and photosynthetic responses of the coccolithophore species *Emiliania huxleyi* under ocean acidification scenarios. We did not locate more than 4 unique articles per crustacean species for exoskeleton structure and function, averaging both datasets. Kroeker et al. (2013) reported 34 total effect sizes for crustaceans, across all surveyed parameters (survival, calcification, growth, development, and abundance). The authors reported higher total numbers of effect sizes for other metazoan marine calcifying taxa: 46 for corals, 53 for echinoderms, and 81 for molluscs. In contrast to our approach, the “crustacean” subset in Kroeker et al. (2013) also included planktonic species. After applying our second inclusion criteria (that excluded copepods, which do not produce mineralized exoskeletons), we removed 51 articles from the previous subset of 179, or 28.5% of the articles at that step. Thus, compared with metazoan taxa considered more sensitive to the effects of climate change (corals, echinoderms, and mollusks), crustaceans are underrepresented in the literature. Although beyond the scope of this study, it is worth noting that unicellular marine calcifiers sensitive to OA, such as the ecologically important foraminifera (Moy et al., 2009; Prazeres et al., 2015), also contributed few (4) effect sizes to Kroeker et al. (2013).

Our quantity of included articles is comparable with reviews on other mobile marine taxa under ocean acidification scenarios. Bednaršek et al. (2019) identified 15 articles on pteropod calcification under ocean acidification. Cattano et al. (2018) summated 25 articles on fish physiology under ocean acidification, a surprisingly low number for a relatively diverse group of organisms. While non‐planktonic crustaceans are still understudied compared with other sessile marine calcifiers, their representation in the literature is comparable with other taxonomic groups.

Our systematic review, comprehensive for English‐language publications, does not encompass literature published in non‐English journals. The limitations of this approach are revealed through biases in subgroup designations, such as the large amount of temperate collection sites compared with tropical regions. While this information is important for Western/Northern conservation and aquaculture in Western/Northern countries, comparable ecosystems and social structures in Southern/so‐called “developing” regions are more vulnerable to the effects of ocean acidification, generally due to limited adaptive capacities and resources (Stewart‐Sinclair et al., 2020). Of note was the lack of papers from East Asian authors—by recent estimates, Chinese aquaculture in 2016 accounted for over 56% of all crustacean products globally (Tacon, 2018).

Our review identified that the majority of published, English‐language studies detailing the effects of ocean acidification on crustacean exoskeletal properties focus on the Orders *Decapoda* and *Sessilia*. This focus likely reflects the economic and ecological value of these taxonomic groups, as well as accessibility for laboratory and field studies. Decapods, particularly marine shrimp, crabs, and lobsters, comprise an estimated quarter of all 2018 global aquaculture output (FAO, 2020). As such, decapods are research stalwarts for their economic roles, in addition to a continued use as model systems for physiology, neurobiology, and toxicology (Knigge et al., 2021; Paul et al., 1985). Barnacles enjoy a history of research on their ecology, physiology, and development, starting with Charles Darwin's set of groundbreaking monographs (Darwin, 1854), and continuing through to modern studies on their role in biofouling communities. However, marine isopods, which similarly mineralize their exoskeletons with CaCO_3_, albeit usually in amorphous forms (Neues et al., 2007; Wood & Russell, 1987), have received almost no attention within the context of ocean acidification.

### 4.2 Meta‐analysis summary

Table 3 summarizes the direction of change and statistical significance of summary effect sizes calculated in our study. Our meta‐analysis revealed complex trends in the mineralogical responses (exoskeleton Ca^2+^ and Mg^2+^ content) of decapods and barnacles to increased seawater *p*CO_2_. The functional properties of the exoskeleton displayed statistically significant decreases (mechanical properties), or a non‐significant tendency to decrease (thickness), under low and moderately high *p*CO_2_. The trends toward decreasing exoskeleton ionic content, hardness, and thickness are interwoven with significant heterogeneity between individual effect sizes (*Q_E_*) for each subgrouping and parameter. Strikingly, Ca^2+^ and Mg^2+^ dynamics tended to co‐vary predictably under CO_2_ regimes less than 1500 µatm, but not higher *p*CO_2_ scenarios.

**TABLE 3:** Summary of results from meta‐analysis. Magnitude of summary effect size is indicated as either positive (+), negative (−), or no change (=). Effect sizes significantly different from 0 are highlighted in yellow

Parameter	*p*CO_2_ range (µatm)			
	500–999	1000–1499	1500–1999	2000+
Calcium	+	+	−	−
Magnesium	−	+	−	+
Ca:Mg	−	+	=	=
Biomechanics	−	ND	−	=
Thickness	−	−	−	=

ND, no data available for subgroup.

### 4.3 Comparisons with results of previous meta‐analyses

Comparable with results from other meta‐analyses, our study indicated that crustacean exoskeleton biomineralization under lower influxes of CO_2_ (<1000 µatm) is relatively stable compared to ambient conditions. Kroeker et al. (2013) noted no differences in crustacean calcification effect sizes under end‐of‐century acidification prediction scenarios (decrease in pH ≤ 0.5 pH units). Harvey et al. (2013), with a smaller sample size than Kroeker et al. (2013), similarly noted no effects of ocean acidification on crustacean calcification rates compared with ambient conditions. We also observed no significant effects of similar levels of elevated CO_2_ (<1500 µatm) on the Ca^2+^ and Mg^2+^ content of crustacean exoskeletons, although ion levels did show complex tendencies to increase (for Ca^2+^) and increase or decrease (for Mg^2+^) (Figure 2b and c). However, we did observe significant negative effects of lower *p*CO_2_ regimes on exoskeletal biomechanics (Figure 4a), a metric not included in either of the earlier studies. Additionally, neither Kroeker et al. (2013) nor Harvey et al. (2013) examined effect sizes for studies with acidification levels beyond the then‐current IPCC ocean pH predictions for 2100 (decrease in pH units >0.5 pH units, *p*CO_2_ influxes of 1500+ µatm). Our study fills this gap, suggesting that crustacean calcification is negatively affected at higher *p*CO_2_ levels at the structural (Figure 2b and c) and functional (Figure 4a) levels. Generally, our results are congruous with other meta‐analyses that assessed a wider range of *p*CO_2_ conditions (500–2000+ µatm), wherein higher CO_2_ levels trended with decreased fitness (survival and reproduction) in crustaceans (Wittmann & Portner, 2013) and shell integrity in invertebrates generally (Hancock et al., 2020).

### 4.4 Meta‐analysis: ion content and biomechanics

Ion content can alter the function and performance of the crustacean exoskeleton. Addition of Mg^2+^ to CaCO_3_, such as the Mg‐calcite polymorph, augments material stability and strength even with minimal incorporation of Mg^2+^ (Kunitake et al., 2012; Long et al., 2014). Accordingly, some studies indicate that high‐use exoskeletal structures, such as the decapod chelae, can possess higher levels of Mg^2+^ than other anatomical regions (Mergelsberg et al., 2019; Page et al., 2017). This trend shows species specificity, such as comparable Mg levels observed in both the carapace and chelae of the swimming crab *Necora puber* (Small et al., 2010). Increased exoskeletal Ca^2+^ levels, however, do not presume greater biomechanical resistance. For example, while the major chelae of the boreoarctic crab *Cancer borealis* has significantly higher Ca^2+^ enrichment than chelae of the crab *Paralomis birsteini*, the microhardness of the former is significantly lower than the latter (Steffel et al., 2019). Functional differences between crustacean exoskeletons could arise from divergent absolute levels of Ca^2+^, Mg^2+^, and their ratio. Other foci important in understanding the mechanisms of ocean acidification‐mediated changes in exoskeletal properties include structural changes in the orientation of crystals within the mineralized layer (Chen et al., 2008; Rosen et al., 2020) and characterization of mineral deposition versus dissolution rates (Bednaršek et al., 2021), facets we did not assess in this study.

Our results suggest exoskeleton Ca^2+^ and Mg^2+^ dynamics vary between high *p*CO_2_ regimes (Table 3). Within the 500–999 µatm range, Ca^2+^ and Mg^2+^ levels tended to increase and decrease, respectively. Differential regulation of ionic content at the lowest *p*CO_2_ range suggests a trade‐off: minimizing Mg^2+^ content decreases exoskeleton strength (Elsternova et al., 2010) but limits potential exoskeleton dissolution common to Mg‐reinforced CaCO_3_ (Andersson et al., 2008). While neither the Ca^2+^ nor Mg^2+^ summary effect sizes under 500–999 µatm *p*CO_2_ were statistically different than 0, their inverse relationship was statistically significant (Figure 3). This suggests that, while relative levels of exoskeletal Ca^2+^ and Mg^2+^ might not significantly change under low *p*CO_2_ regimes, their ratio could predictably change to maximize exoskeletal longevity at the cost of exoskeleton function.

Reduction of Mg^2+^ under 500–999 µatm stands in contrast to the tendency for both Ca^2+^ and Mg^2+^ to increase under a 1000–1499 µatm regime. Previous experimental studies noted positive effects of moderate *p*CO_2_ (i.e., the ‘business as usual’ model of ocean acidification) on crustacean calcification (McDonald et al., 2009; Ries et al., 2009), a result often attributed to crustaceans’ ability to utilize influxes of ${\text{HCO}}_{3}^{‐}$ during shell formation (Cameron & Wood, 1985; Whiteley, 2011). In line with this hypothesis, the relationship between changes in Ca^2+^ and Mg^2+^ levels under 1000–1499 µatm showed a significant positive relationship (Figure 3), suggesting some of the crustacean species assayed under 1000–1499 µatm can increase their mineralization with moderate influxes of CO_2_. However, the statistically significant *Q_E_* value for this *p*CO_2_ range indicated substantial heterogeneity between crustacean responses (Tables A1–A6). Thus, while some crustaceans may be able to take advantage of increased ${\text{HCO}}_{3}^{‐}$ for mineralization, this ability varies considerably between species and studies and likely depends on a wide range of other physiological factors.

We noted significant decreases in both Ca^2+^ and Mg^2+^ content under moderately high *p*CO_2_, 1500–1999 µatm. The observed decreases were seemingly driven by data from Page et al. (2017): removal of Ca^2+^ and Mg^2+^ data points for the four crab species assayed in the study returned a negative, but non‐significant summary effect size. Biomechanical properties also significantly decreased under 1500–1999 µatm albeit without any measurements from Page et al. (2017). This combination parsimoniously suggests that decreases in Mg‐loaded CaCO_3_, whether through decreased Mg^2+^ uptake or increased Mg^2+^ dissolution, could underlie diminished exoskeletal durability (Dillaman et al., 2005; Findlay et al., 2010; Nardone et al., 2018). Alternatively, the observed decrease in biomaterial strength and hardness could also be driven by alterations in the properties of the organic matrix or organic inclusions within the CaCO_3_ lattice, which can augment biomechanical resistance (Kim et al., 2016; Nardone et al., 2018; Taylor et al., 2015). Further characterization of ocean acidification's effects on the relationship between the organic and inorganic components of the crustacean exoskeleton, as well as prospective chemical changes to the fluids at the site of calcification necessary for CaCO_3_ precipitation (Cameron & Wood, 1985; Reymond & Hohn, 2021), are needed.

Most effect sizes included in the biomechanics dataset were obtained from measurements of exoskeletal microhardness (16 out of 19 effect sizes, ~84%). As such, the negative relationship between Ca^2+^ and Mg^2+^ under 500–999 µatm underlie localized changes in mechanical properties at the tens of microns scale. Micron‐scale hardness testing typically scales with “whole‐shell” mechanical assessments (Currey & Brear, 1990), but cuticle thickness and geometry will also affect these responses. Previous changes in whole exoskeleton biomechanics of anatomical regions used for predation, feeding, or conflict, such as the highly mineralized decapod chelae (deVries et al., 2021; Fabritius et al., 2016; Luquet, 2012), pose direct ecological risks to crustaceans. Further mesocosm and field experiments are needed to determine whether crustaceans exposed to high *p*CO_2_ seawater for set amounts of time experience different ecological outcomes based on their exoskeleton stability, such as in conflict resolution with other organisms (deVries et al., 2021). Application of our results in these experiments is particularly crucial for management of wild‐caught crustacean species already vulnerable to population decline, such as economically important red king crabs and Tanner crabs (Armstrong et al., 1998; Seung et al., 2015).

Decreases in Ca^2+^ content, Mg^2+^ content, and hardness mediated by higher *p*CO_2_ levels indicate that even crustaceans cannot effectively regulate biomineralization under elongated exposure to high *p*CO_2_ conditions. While osmoregulatory capacities vary between crustacean species (Whiteley, 2011), one potential hypothesis for our observed negative summary effect sizes lies in crustacean use of exoskeletal ${\text{HCO}}_{3}^{‐}$ for acid‐base regulation. Experimental evidence from decapod species provides some support for HCO_3_‐mediated buffering under acidification scenarios. After 24 h of exposure to CO_2_‐induced hypercapnia, increased hemolymph levels of ${\text{HCO}}_{3}^{‐}$, but not Ca^2+^ or Mg^2+^, were observed in the blue crab *Callinectes sapidus* (Cameron & Iwama, 1987). In a comparative study, the prawns *Palaemon elegans* and *Palaemon serratus* differentially altered hemolymph ion content throughout 30 days of CO_2_‐induced hypercapnia. Ca^2+^ levels significantly differed after 14 days of hypercapnia but returned to levels of ambient‐exposed animals after 30 days for both species. Mg^2+^ levels, meanwhile, significantly changed after 5 days of hypercapnia for both species but returned to ambient levels when remeasured on study day 14 and remained at this level until study day 30 (Dissanayake et al., 2010). While neither study tested structural or functional properties of crab or prawn exoskeletons following hypercapnic exposure, it is possible that increases in circulating ${\text{HCO}}_{3}^{‐}$, Ca^2+^, and/or Mg^2+^ levels arose from exoskeleton dissolution and, ultimately, would explain the negative effect sizes noted in our results. Studies on velvet swimming crabs (*Necora puber*) exposed to CO_2_‐mediated hypercapnia over 16 days noted increased hemolymph ${\text{HCO}}_{3}^{‐}$, with experimental confirmation that the ions were at least partially supplied by exoskeleton dissolution (Spicer et al., 2006). In the face of ocean acidification, crustaceans face an immediate physiological trade‐off: alter the chemical structure of their exoskeletons (potentially changing functionality) to maintain acid‐base status or maintain exoskeleton structure and integrity at the expense of internal acidosis.

It is important to note that heightened CO_2_ exposure for the three studies discussed above (Cameron & Iwama, 1987; Dissanayake et al., 2010; and Spicer et al., 2006) occurred on a relatively short timescale of days to weeks. Changes in ${\text{HCO}}_{3}^{‐}$, Ca^2+^, or Mg^2+^ levels supplied by exoskeletal dissolution are ostensibly part of an immediate stress response to acidification. However, experimental modeling of long‐term acidification scenarios, and delineation between acute and chronic physiological acclimation, has been a longstanding concern in the field (Kelly & Hofmann, 2012; Whiteley, 2011; Widdicombe & Spicer, 2008). Physiological differences between acute and chronic responses suggest that a quintessential dose‐response framework would not fit *p*CO_2_‐driven responses on the longer, ecologically and evolutionarily relevant timescales needed to properly model acclimation, adaptation, and phenotypic plasticity to climate change scenarios (Stillman & Paganini, 2015).

Recent meta‐analyses on other physiological and life history traits (growth, fecundity, hatching success, and respiration) in decapod crustaceans confirm this difference between acute and chronic acclimation and strongly suggest that prolonged exposure to ocean acidification conditions further exacerbates negative physiological effects (Bednaršek et al., 2021). Definition of organismic responses at the acute versus chronic levels to climate change of any magnitude will require future studies to use physiologically and ecologically relevant timescales for their exposures. This is particularly true of coastal regions that already experience drastic fluctuations in water chemistry on relevant time scales (Baumann et al., 2014) that will only be exacerbated by climate change‐induced modulations of seawater chemistry (Przeslawski et al., 2008; Thomsen et al., 2010). Chronic exposure to high *p*CO_2_ seawater can produce physiological changes that influence biomineralization capacity, such as in Tanner crabs (*Chionoecetes bairdi*) exposed to acidified seawater over 2 years, which showed significant decreases in the internal pH of specialized hemocytes involved in carapace maintenance and molting (Meseck et al., 2016). Indeed, Kroeker et al. (2013) noted that studies of calcification under ocean acidification tended to skew toward the shorter end, with most studies lasting less than 100 days. Extended time‐course experiments, collecting samples during OA exposures, would help to define incremental changes in the chemical and physical attributes of crustacean exoskeletons that can lead to the decreases in exoskeletal Ca^2+^ and Mg^2+^ content and/or strength as described in our study, and potentially identify crustacean species with sublethal vulnerabilities to OA.

### 4.5 Beyond business as usual: multi‐stressor syntheses and response heterogeneity

In this research synthesis, we included manuscripts that only assayed changes in crustacean exoskeleton structure and function under ocean acidification scenarios. However, ocean acidification is only one climate change‐induced alteration to the chemical and physical properties of our oceans (Riebesell & Gattuso, 2015; Sokolova et al., 2016). Crustaceans regularly experience complex variations in seawater pH, salinity, oxygen content, and temperature on multiple time scales. These fluctuations will be further exacerbated by climate change, potentially to detrimental results on marine organisms (see Gunderson et al., 2016 for a comprehensive review on the topic). A growing body of the literature reports the effects and potential synergies of multiple climate change stressors on crustacean growth, development, physiology, and calcification (Lowder et al., 2017; Manriquez et al., 2021; Ramaglia et al., 2018; Walther et al., 2010). The effects observed in these studies must also be reviewed and synthesized, as previously done in the broad taxonomic analyses of Kroeker et al. (2013) and Harvey et al. (2013).

Meta‐analyses on the multi‐stressor effects on crustacean physiology, exoskeletal or otherwise, must be thoughtfully subdivided due to high variability in crustacean responses. Indeed, experimental observations on the effects of multiple stressors on crustacean physiology and biomineralization suggest species and population specific changes. For example, experiments using acorn barnacles (*Amphibalanus improvisus*) collected from Kiel Fjord, Germany revealed negative effects on survival and growth from increased temperature, but not acidification nor the combination thereof (Pansch et al., 2012). Experiments with the Sweden‐collected lobster *N*. *norvegicus* (Order = Decapoda) suggest that embryonic physiological parameters are distinctly perturbed by acidification or warming but not their overlap (Styf et al., 2013). More intricate experiments exacerbating diurnal fluctuations in seawater pH and temperature suggest negative, synergistic effects of acidification and warming on organismic performance in the porcelain crab *Petrolisthes cinctipes* (Paganini et al., 2014). These studies do not examine the effects of multiple climate change stressors on crustacean exoskeleton structure and function, an important facet to assess given potential changes in exoskeleton composition and dissolution with changes in aragonite saturation states concomitant with increased *p*CO_2_ and temperature (Fabritius et al., 2016).

Our analysis strongly indicates high heterogeneity between‐study effect sizes within each *p*CO_2_ regime, as evidenced by large, statistically significant *Q* values for most structural and functional properties (Tables A3–A8). Increases in the variance of exoskeleton properties under ocean acidification, particularly structural parameters, have been noted for crustaceans in both *Decapoda* and *Sessilia*. Carapace samples from grass shrimp (*Lysmata californiaca*) exposed to acidified seawater contain, on average, comparable levels of Ca^2+^ to ambient‐exposed animals, but higher variation than ambient (Lowder et al., 2017). Similarly, cold‐water barnacles (*Balanus improvisus*) exposed to fluctuating or constantly acidified seawater build shells with ambient‐comparable levels of Ca^2+^ but with greater variance, particularly in fluctuating acidification conditions (Eriander et al., 2016). The source of the heterogeneity remains elusive, and difficult to relate between physiological studies conducted on individuals, as summarized in our study, and broader genetic studies of multiple species populations. Population studies indicate that environmental effects produce stronger effects on population‐level variability in decapod morphology than genetical structure (Brian et al., 2006). However, recent genetic studies suggest that barnacle populations, while sessile and subject to high mortality and dispersal of juveniles, harbor considerable functional genetic variation in metabolic genes, maintained by strong balancing selection from their highly variable habitats (Nunez et al., 2021). If increased variance in crustacean exoskeleton properties is a common occurrence under ocean acidification, then future studies must take care to incorporate this population‐ or individual‐specific variability into their experimental design. Future mechanistic studies on crustacean mineralization under OA should include comparative approaches between animals of the same species inhabiting different regions with different physical and chemical seawater properties, or different species co‐inhabiting the same area but differentially responding to OA, with goals to identify genetic variance underlying OA resilience or susceptibility and resultant biochemical and material markers. Ultimately, if scientists wish to understand how climate change will impact crustaceans on any level of biological organization, we must integrate crustacean diversity into our experiments, interpretations, and recommendations.

## CONFLICT OF INTEREST

The authors declare no conflicts of interests in the publication of this study.

### AUTHOR CONTRIBUTION

**Kyle R. Siegel:** Conceptualization (equal); Data curation (lead); Formal analysis (lead); Investigation (lead); Methodology (lead); Project administration (lead); Resources (equal); Software (lead); Validation (lead); Visualization (lead); Writing – original draft (lead); Writing – review & editing (equal). **Muskan Kaur:** Data curation (equal); Investigation (equal); Writing – review & editing (equal). **A. Calvin Grigal:** Data curation (equal); Investigation (equal); Writing – review & editing (equal). **Rebecca A. Metzler:** Funding acquisition (equal); Methodology (equal); Writing – review & editing (equal). **Gary H. Dickinson:** Conceptualization (equal); Funding acquisition (lead); Methodology (equal); Project administration (equal); Resources (equal); Supervision (lead); Writing – review & editing (equal).

## Supporting information

Supplementary Material

Supplementary Material

## Data Availability

All Excel files, CSV files, and R code can be found on Dryad (DOI https://doi.org/10.5061/dryad.x3ffbj7mv).
