# Supplementary material for: Meta‐analysis suggests negative, but pCO2‐specific, effects of ocean acidification on the structural and functional properties of crustacean biomaterials
Source: Ecol Evol. 2022 Jun 3;12(6):e8922. doi: 10.1002/ece3.8922 (PMC9165209; doi:10.1002/ece3.8922)
Supplement: Supplementary file 1 — Supplementary Material [file ECE3-12-e8922-s002.docx]

**Supplementary Materials to Siegel et al. 2022**

**Supplementary Table 1. Google Scholar Profiles Assessed During Systematic Review**

| **Researcher name** | **Google Scholar URL** |
| --- | --- |
| Helen Findlay |  |
| Kurihara, Haruko |  |
| Long, William Christopher |  |
| Otto-Portner, Hans |  |
| Ries, Justin |  |
| Small, Daniel |  |
| Spicer, John |  |
| Stillman, Jonathon |  |
| Taylor, Jennifer R.A. |  |
| Wahl, Martin |  |
| Widdicombe, Stephen |  |

**Supplemental Document 1. Equations Used for Standard Deviation Conversions**

For standard error (SE): $SD= \sqrt{n}*SE$

where n is the sample size for a group and SE is the standard error

For 95% confidence intervals: $SD=(\sqrt{n}*\left( upper limit-lower limt \right))/3.92$

For 25^th^ and 75^th^ quartiles in an interquartile range:  $SD= \frac{X- \mu}{Z}$

where µ is the mean of the group, *x* is the interquartile range, and *z* is the Χ^2^ distribution value at that quartile. Here, we obtained SD values from the 25^th^ and 75^th^ interquartile ranges and used their average for each effect size.

**Supplementary Document 2. Conversion equations to** $\frac{\boldsymbol{\mu mol}}{\boldsymbol{mg}}$ **for ionic content:**

$$\frac{\boldsymbol{mmol}}{\boldsymbol{L}}\boldsymbol{\times}\frac{\boldsymbol{1} \boldsymbol{mol}}{\boldsymbol{1} \boldsymbol{mmol}}\boldsymbol{\times}\frac{\boldsymbol{0}\boldsymbol{.}\boldsymbol{001} \boldsymbol{L}}{\boldsymbol{1} \boldsymbol{g}}\boldsymbol{\times}\frac{\boldsymbol{1000000} \boldsymbol{\mu mol}}{\boldsymbol{1} \boldsymbol{mol}}\boldsymbol{\times}\frac{\boldsymbol{1} \boldsymbol{g}}{\boldsymbol{1000} \boldsymbol{mg}}\boldsymbol{=}\frac{\boldsymbol{\mu mol}}{\boldsymbol{mg}}$$

$$\frac{\boldsymbol{\mu mol}}{\boldsymbol{g}}\boldsymbol{\times}\frac{\boldsymbol{1} \boldsymbol{g}}{\boldsymbol{1000} \boldsymbol{mg}}\boldsymbol{=}\frac{\boldsymbol{\mu mol}}{\boldsymbol{mg}}$$

$$\frac{\boldsymbol{mg}}{\boldsymbol{g}}\boldsymbol{\times}\frac{\boldsymbol{1} \boldsymbol{g}}{\boldsymbol{1000} \boldsymbol{mg}}\boldsymbol{\div}{\frac{\boldsymbol{g}}{\boldsymbol{mol}}}^{\boldsymbol{*}}\boldsymbol{\times}\frac{\boldsymbol{1000000} \boldsymbol{\mu mol}}{\boldsymbol{1} \boldsymbol{mol}}\boldsymbol{\times}\frac{\boldsymbol{1} \boldsymbol{g}}{\boldsymbol{1000} \boldsymbol{mg}}\boldsymbol{=}\frac{\boldsymbol{\mu mol}}{\boldsymbol{mg}}$$

$$\frac{\boldsymbol{ng}}{\boldsymbol{g}}\boldsymbol{\times}\frac{\boldsymbol{1} \boldsymbol{g}}{\boldsymbol{1}\boldsymbol{*}\boldsymbol{10}^{\boldsymbol{9}} \boldsymbol{ng}}\boldsymbol{\div}{\frac{\boldsymbol{g}}{\boldsymbol{mol}}}^{\boldsymbol{*}}\boldsymbol{\times}\frac{\boldsymbol{1000000} \boldsymbol{\mu mol}}{\boldsymbol{1} \boldsymbol{mol}}\boldsymbol{\times}\frac{\boldsymbol{1} \boldsymbol{g}}{\boldsymbol{1000} \boldsymbol{mg}}\boldsymbol{=}\frac{\boldsymbol{\mu mol}}{\boldsymbol{mg}}$$

$$\frac{\boldsymbol{mmol}}{\boldsymbol{kg}}\boldsymbol{\times}\frac{\boldsymbol{1} \boldsymbol{kg}}{\boldsymbol{1000000} \boldsymbol{mg}}\boldsymbol{\times}\frac{\boldsymbol{1000} \boldsymbol{\mu mol}}{\boldsymbol{1} \boldsymbol{mol}}\boldsymbol{=}\frac{\boldsymbol{\mu mol}}{\boldsymbol{mg}}$$

$$\frac{\boldsymbol{mmol}}{\boldsymbol{g}}\boldsymbol{\times}\frac{\boldsymbol{1} \boldsymbol{g}}{\boldsymbol{1000} \boldsymbol{mg}}\boldsymbol{\times}\frac{\boldsymbol{1000} \boldsymbol{\mu mol}}{\boldsymbol{1} \boldsymbol{mmol}}\boldsymbol{=}\frac{\boldsymbol{\mu mol}}{\boldsymbol{mg}}$$

*****Conversion by element molecular weight – Ca: 40.078 g/mol; Mg: 24.305 g/mol

**Supplementary Document 3: References for articles included in the meta-analyses**

***Ion concentration***

Arnold, K. E., Findlay, H. S., Spicer, J. I., Daniels, C. L., and Boothroyd, D.: Effect of CO_2_-related acidification on aspects of the larval development of the European lobster, *Homarus gammarus* (L.), Biogeosciences, 6, 1747–1754, https://doi.org/10.5194/bg-6-1747-2009, 2009.

Coffey, W.D., Nardone, J.A., Yarram, A., Long, W.C., Swiney, K.M., Foy, R.J., and Dickinson, G.H. 2017. Ocean acidification leads to altered micromechanical properties of the mineralized cuticle in juvenile red and blue king crabs. *Journal of Experimental Marine Biology and Ecology*, 495:1-12.

Dickinson, G.H., Bejerano, S., Salvador, T., Makdisi, C., Patel, S., Long, W.C., Swiney, K.M., Foy, R.J., Steffel, B.V., Smith, K.E., and Aronson, R.B. 2021. Ocean acidification alters properties of the exoskeleton in adult Tanner crabs, *Chionoectes bairdi*. *Journal of Experimental Biology*, 224(3):jeb232819. doi:https://doi.org/10.1242/jeb,232819

Dickinson, G.H., Nardone, J.A., Patel, S., Siegel, K.R., Tedesco, D., Schultzhaus, J.N., Leary, D.H., Spillmann, C.M., Metzler, R.A., Orihuela, B., and Rittschof, D. 2021b. Interactive Effects of Ocean Acidification and Warming on Shell and Adhesive Properties in the Barnacle *Amphibalanus amphitrite*. In preparation.

Donohue P., Calosi P., Bates A.H., Laverock B, Rastrick, S., Mark, F.C., Strobel, A., and Widdicombe, S. (2012) Impact of exposure to elevated pCO_2_ on the physiology and behaviour of an important ecosystem engineer, the burrowing shrimp *Upogebia deltaura*. Aquat Biol 15:73-86. <https://doi.org/10.3354/ab00408>

Findlay HS, Kendall MA, Spicer JI, Widdicombe S (2009) Future high CO_2_ in the intertidal may compromise adult barnacle *Semibalanus balanoides* survival and embryonic development rate. Mar Ecol Prog Ser 389:193-202. <https://doi.org/10.3354/meps08141>

Findlay, H.S., Kendall, M.A., Spicer, J.I., Widdicombe, S. Post-larval development of two intertidal barnacles at elevated CO_2_ and temperature. *Mar Biol* **157,**725–735 (2010). https://doi.org/10.1007/s00227-009-1356-1

Findlay, H.S., Kendall, M.A., Spicer, J.I., and Widdicombe, S. 2010. Relative influences of ocean acidification and temperature on intertidal barnacle post-larvae at the northern edge of their geographic distribution. *Estuarine, Coastal and Shelf Science*, 86(4):675-682. https://doi.org/10.1016/j.ecss.2009.11.036

Glandon, H.L., Kilbourne, K.H., Schijf, J., and Miller, T.J. 2018. Counteractive effects of increased temperature and *p*CO_2_ on the thickness and chemistry of the carapace of juvenile blue crab, *Callinectes sapidus*, from the Patuxent River, Chesapeake Bay. *Journal of Experimental Marine Biology and Ecology*, 498:39-45.

Long, W.C., Swiney, K.M., and Foy, R.J. 2013a. Effects of ocean acidification on the embryos and larvae of red king crab, *Paralithodes camtschaticus*. *Marine Pollution Bulletin*, 69(1-2):38-47.

Long, W.C., Swiney, K.M., Harris, C., Page, H.N., and Foy, R.J. 2013b. Effects of Ocean Acidification on Juvenile Red King Crab (*Paralithodes camtschaticus*) and Tanner Crab (*Chionoecete bairdi*) Growth, Condition, Calcification, and Survival. *PLoS One*, doi: https://doi.org/10.1371/journal.pone.0060959

Lowder, K.B., Allen, M.C., Day, J.M.D., Deheyn, D.D., and Taylor, J.R.A. 2017. Assessment of ocean acidification and warming on the growth, calcification, and biophotonics of a California grass shrimp. *ICES Journal of Marine Science*, 74(4):1150-1158. doi: https://doi.org/10.1093/icesjms/fws246

Menu-Courey, K., Noisette, F., Piedalue, S., Daoud, D., Blair, T., Blier, P.U., Azetsu-Scott, K., and Calosi, P. 2019. Energy metabolism and survival of the juvenile recruits of the American lobster (*Homarus americanus*) exposed to a gradient of elevated seawater *p*CO_2_. *Marine Environmental Research*, 143:111-123.

Nardone, J.A., Patel, S., Siegel, K.R., Tedesco, D., McNicholl, C.G., O’Malley, J., Herrick, J., Metzler, R.A., Orihuela, B., Rittschof, D., and Dickinson, G.H. 2018. Assessing the Impacts of Ocean Acidification on Adhesion and Shell Formation in the Barnacle *Amphibalanus amphitrite*. *Frontiers in Marine Science*, doi: https://doi.org/10.3389/fmars.2018.00369

Page, T., Worthington, S., Calosi, P., and Stillman, J.H. 2017. Effects of elevated *p*CO_2_ on crab survival and exoskeleton composition depend on shell function and species distribution: a comparative analysis of carapace and claw mineralogy across four porcelain crab species from different habitats. *ICES Journal of Marine Science*, 74(4):1021-1032.

Rankin, A., Seo, K., Graeve, O.A., and Taylor, J.R.A. 2019. No compromise between metabolism and behavior of decorator crabs in reduced pH conditions. *Scientific Reports*, 9:6262.

Small D., Calosi P., White D, Spicer J.I., and Widdicombe S. (2010) Impact of medium-term exposure to CO_2_ enriched seawater on the physiological functions of the velvet swimming crab *Necora puber*. Aquat Biol 10:11-21. <https://doi.org/10.3354/ab00266>

Small, D.P. Calosi, P., Boothroyd, D., Widdicombe, S., and Spicer, J.I. 2015. Stage-Specific Changes in Physiological and Life-History Responses to Elevated Temperature And PCO_2_ during the Larval Development of the European Lobster *Homarus Gammarus* (L.). *Physiological and Biochemical Zoology*, 88(5): 494-507.

Small, D.P., Calosi, P., Boothroyd, D., Widdicombe, S., and Spicer, J.I. 2016. The sensitivity of the early benthic juvenile stage of the European lobster *Homarus Gammarus* (L.) to elevated *p*CO_2_ and temperature. *Marine Biology*, 163(53).

Swiney, K.M., Long, W.C., and Foy, R.J. 2016. Effects of high *p*CO_2_ on Tanner crab reproduction and early life history – Part I: long-term exposure reduces hatching success and female calcification, and alters embryonic development. *ICES Journal of Marine Science*, 73(3):825-835.

Taylor, J.R.A., Gilleard, J.M., Allen, M.C., and Deheyn, D.D. 2015. Effects of CO_2_-induced pH reduction on the exoskeleton structure and biophotonic properties of the shrimp *Lysmata californica*. *Scientific Reports*, 5:10608.

Turra, A., Ragagnin, M.N., McCarthy, I.D., and Fernandez, W.S. 2020. The effect of ocean acidification on the intertidal hermit crab *Pagarus crinitcornis* is not modulated by cheliped amputation and sex. *Marine Environmental Research*, 153:104794.

***Biomechanical properties and total cuticle thickness***

Coffey, W.D., Nardone, J.A., Yarram, A., Long, W.C., Swiney, K.M., Foy, R.J., and Dickinson, G.H. 2017. Ocean acidification leads to altered micromechanical properties of the mineralized cuticle in juvenile red and blue king crabs. *Journal of Experimental Marine Biology and Ecology*, 495:1-12.

Dickinson, G.H., Bejerano, S., Salvador, T., Makdisi, C., Patel, S., Long, W.C., Swiney, K.M., Foy, R.J., Steffel, B.V., Smith, K.E., and Aronson, R.B. 2021. Ocean acidification alters properties of the exoskeleton in adult Tanner crabs, *Chionoectes bairdi*. *Journal of Experimental Biology*, 224(3):jeb232819. doi:https://doi.org/10.1242/jeb,232819

Dickinson, G.H., Nardone, J.A., Patel, S., Siegel, K.R., Tedesco, D., Schultzhaus, J.N., Leary, D.H., Spillmann, C.M., Metzler, R.A., Orihuela, B., and Rittschof, D. Unpublished (as of 28Jan2022). Interactive Effects of Ocean Acidification and Warming on Shell and Adhesive Properties in the Barnacle *Amphibalanus amphitrite*. In preparation.

Eriander, L., Wrange, A.-L., and Havenhand, J.N. 2016. Simulated diurnal pH fluctuations radically increase *variance* in – but not the *mean* of – growth in the barnacle *Balanus improvisus*. *ICES Journal of Marine Science*, 73(3):596-603.

Glandon, H.L., Kilbourne, K.H., Schijf, J., and Miller, T.J. 2018. Counteractive effects of increased temperature and *p*CO_2_ on the thickness and chemistry of the carapace of juvenile blue crab, *Callinectes sapidus*, from the Patuxent River, Chesapeake Bay. *Journal of Experimental Marine Biology and Ecology*, 498:39-45.

Nardone, J.A., Patel, S., Siegel, K.R., Tedesco, D., McNicholl, C.G., O’Malley, J., Herrick, J., Metzler, R.A., Orihuela, B., Rittschof, D., and Dickinson, G.H. 2018. Assessing the Impacts of Ocean Acidification on Adhesion and Shell Formation in the Barnacle *Amphibalanus amphitrite*. *Frontiers in Marine Science*, doi: https://doi.org/10.3389/fmars.2018.00369

Pansch, C., Schaub, I., Havenhand, J., and Wahl, M. 2013. Habitat traits and food availability determine the response of marine invertebrates to ocean acidification. *Global Change Biology*, 20(3):765-777.

Taylor, J.R.A., Gilleard, J.M., Allen, M.C., and Deheyn, D.D. 2015. Effects of CO_2_-induced pH reduction on the exoskeleton structure and biophotonic properties of the shrimp *Lysmata californica*. *Scientific Reports*, 5:10608.
