# Supplementary material for: Meta‐analysis suggests negative, but pCO2‐specific, effects of ocean acidification on the structural and functional properties of crustacean biomaterials
Source: Ecol Evol. 2022 Jun 3;12(6):e8922. doi: 10.1002/ece3.8922 (PMC9165209; doi:10.1002/ece3.8922)
Supplement: Supplementary file 2 — Supplementary Material [file ECE3-12-e8922-s001.docx]

**Appendix**


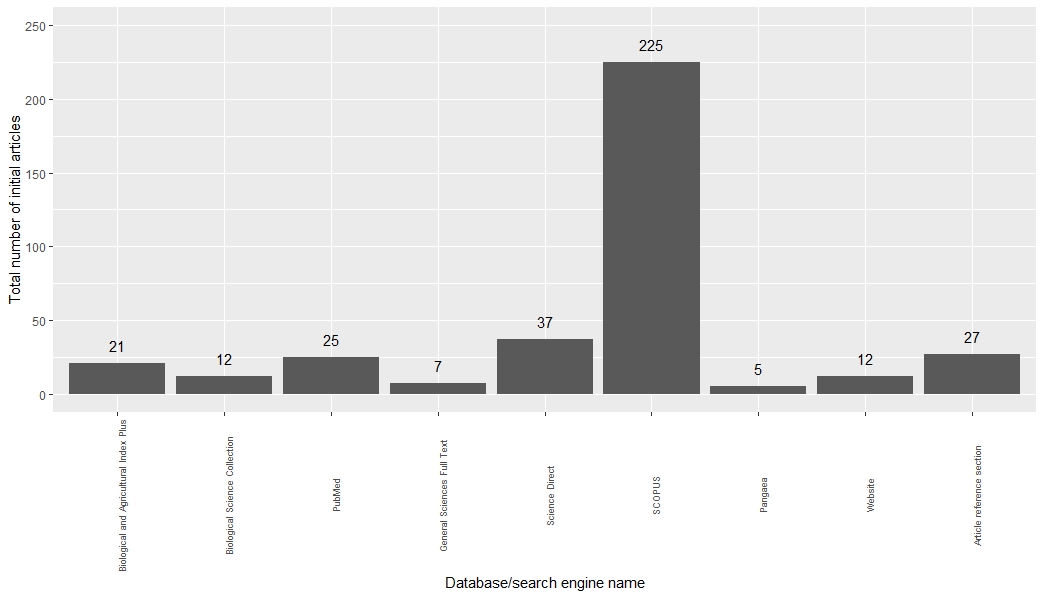


**Appendix Figure 1.** Number of articles that met the primary inclusion criteria among databases and search engines during the systematic review. Among search platforms used, SCOPUS returned more potential hits than any other engine, followed by Science Direct.

**Appendix Table 1**. Articles included in the biomechanical dataset for measurements of exoskeleton Ca^2+^ and Mg^2+^ concentrations. Numbers in parentheses next to ‘Yes’ or ‘No’ indicate number of data points extracted for that parameter, from that paper.

| Article  shorthand | Species | Taxonomy | Measure  method | Calcium | Magnesium |
| --- | --- | --- | --- | --- | --- |
| Arnold et al.  2009 | *Homarus gammarus* | Decapoda | ICP | Yes (1) | Yes (1) |
| Coffey et al.  2017 | *Paralithodes platypus* | Decapoda | SEM/EDS | Yes (2) | Yes (2) |
|  | *Paralithodes camtschaticus* | Decapoda | SEM/EDS | Yes (1) | Yes (1) |
| Dickinson et al. (unpublished) | *Amphibalanus amphitrite* | Sessilia | SEM/EDS | Yes (2) | Yes (2) |
| Dickinson et al. 2021 | *Chionoecetes bairdi* | Decapoda | ICP-OES | Yes (4) | Yes (4) |
| Donohue et al. 2012 | *Upogebia deltaura* | Decapoda | ICP-OES | Yes (4) | Yes (4) |
| Findlay et al. 2009 | *Semibalanus belanoides* | Sessilia | ICP-OES | Yes (1) | No |
|  | *Elminius modestus* | Sessilia | ICP-OES | Yes (1) | No |
| Findlay et al. 2010 | *Semibalanus belanoides* | Sessilia | ICP-OES | Yes (1) | Yes (1) |
| Findlay et al. 2011 | *Semibalanus belanoides* | Sessilia | ICP | Yes (2) | Yes (1) |
| Glandon et al. 2018 | *Callinectes sapidus* | Decapoda | ICP-AES | Yes (1) | No |
| Long et al. 2013a | *Paralithodes*  *camtschaticus* | Decapoda | Ion chromatography | Yes (1) | Yes (1) |
| Long et al. 2013b | *Chionoecetes bairdi* | Decapoda | Ion chromatography | Yes (2) | No |
|  | *Paralithodes camtschaticus* | Decapoda | Ion chromatography | Yes (1) | No |
| Lowder et al. 2017 | *Hippolyte californiensis* | Decapoda | ICP-MS | Yes (1) | Yes (1) |
| Menu-Courey et al. 2019 | *Homarus americanus* | Decapoda | MS-ICP-MS | No | Yes (2) |
| Nardone et al. 2018 | *Amphibalanus amphitrite* | Sessilia | SEM/EDS | Yes (4) | Yes (4) |
| Page et al. 2017 | *Petrolisthes cinctipes* | Decapoda | ICP-MS | Yes (2) | Yes (2) |
|  | *Petrolisthes manimaculis* | Decapoda | ICP-MS | Yes (2) | Yes (2) |
|  | *Porcellana platycheles* | Decapoda | ICP-MS | Yes (2) | Yes (2) |
|  | *Pisidia longicornis* | Decapoda | ICP-MS | Yes (1) | Yes (1) |
| Rankin et al. 2019 | *Pelia tumida* | Decapoda | SEM or ICP-MS | Yes (1) | Yes (1) |
| Small et al. 2010 | *Necora puber* | Decapoda | AAS | Yes (4) | Yes (4) |
| Small et al. 2015 | *Homarus gammarus* | Decapoda | ICP-OES | Yes (1) | Yes (1) |
| Small et al. 2016 | *Homarus gammarus* | Decapoda | ICP-OES | Yes (1) | Yes (1) |
| Swiney et al. 2016 | *Chionoecetes bairdi* | Decapoda | **unknown** | Yes (2) | Yes (2) |
| Taylor et al. 2015 | *Lysmata californica* | Decapoda | SEM/EDS | Yes (1) | Yes (1) |
| Turra et al. 2020 | *Pagurus criniticornis* | Decapoda | flame AAS | Yes (2) | No |
| AAS, atomic absorption spectrometry; AES, atomic emission spectroscopy; EDS, electron dispersive spectroscopy; ICP, inductively coupled plasma; MS, mass spectrometry; OES, optical emission spectrometry; SEM, scanning electron microscopy | | | | | |

**Appendix Table 2**. Articles included in the biomechanical dataset for measurements of structure hardness and thickness. Numbers in parentheses next to ‘Yes’ or ‘No’ indicate number of data points extracted for that parameter, from that paper.

| Article  shorthand | Species | Taxonomy | Biomech. | Measure  method | Cuticle thickness | Measure method | |
| --- | --- | --- | --- | --- | --- | --- | --- |
| Coffey et al 2017 | *Paralithodes platypus* | Decapoda | Yes (4) | Micro-  indentation | Yes (4) | Light micro-scopy | |
|  | *Paralithodes camtschaticus* | Decapoda | Yes (2) |  | Yes (2) |  |  |
| Dickinson et al 2021 | *Chionoecetes bairdi* | Decapoda | Yes (4) | Micro-  indentation | Yes (4) | Light micro-scopy | |
| Dickinson et al (unpublished) | *Amphibalanus amphitrite* | Sessilia | Yes (2) | Micro-  indentation | Yes (2) | Light micro-scopy | |
| Eriander et al 2015 | *Balanus improvisus* | Sessilia | Yes (1) | Force  gauge | No | NA | |
| Glandon et al 2018 | *Callinectes sapidus* | Decapoda | No | NA | Yes (1) | Light micro-scopy | |
| Nardone et al 2018 | *Amphibalanus amphitrite* | Sessilia | Yes (4) | Microindentation | Yes (4) | Light micro-scopy | |
| Pansch et al. 2014 | *Amphibalanus improvisus* | Sessilia | Yes (2) | Texture  analyzer | No | NA | |
| Taylor et al. 2015 | *Lysmata californica* | Decapoda | No | NA | Yes (1) | SEM | |
| NA, not applicable; SEM, scanning electron microscopy | | | | | | |  |

**Appendix Table 3. Q values for *p*CO_2_ ranges in the combined ion (Ca**^2+^ **and Mg**^2+^**) analysis**

| Combined (Ca and Mg) model results | | | | |
| --- | --- | --- | --- | --- |
| *p*CO_2_ range (µatm) | Q | df | p-value | Model direction |
| 500-999 | 69.8895 | 29 | < .0001 | No effect |
| 1000-1499 | 91.5321 | 20 | < .0001 | No effect |
| 1500-1999 | 86.3847 | 28 | < .0001 | Negative |
| 2000+ | 46.2048 | 15 | < .0001 | No effect |

**Appendix Table 4. Q values for *p*CO_2_ ranges in the Ca**^2+^ **analysis**

| Ca model results | | | | |
| --- | --- | --- | --- | --- |
| *p*CO_2_ range (µatm) | Q | df | p-value | Model direction |
| 500-999 | 30.4165 | 15 | 0.0105 | No effect |
| 1000-1499 | 73.4051 | 11 | < .0001 | No effect |
| 1500-1999 | 55.7199 | 14 | < .0001 | Negative |
| 2000+ | 11.7517 | 7 | **0.1090** | No effect |

**Appendix Table 5. Q values for *p*CO_2_ ranges in the Ca**^2+^ **by taxonomic order analysis**

| Calcium: Taxonomic order | | | | | | |
| --- | --- | --- | --- | --- | --- | --- |
| *p*CO_2_ range | Q_e_ | | | Q_m_ | | |
|  | Q_e_ | df | p-value | Q_m_ | df | p-value |
| Overall | 179.2602 | 49 | < .0001 | 5.51232 | 2 | 0.0772 |
| 500-999 | 29.6581 | 14 | 0.0085 | 0.3814 | 2 | 0.8264 |
| 1000-1499 | 73.405 | 10 | < .0001 | 0.5005 | 2 | 0.7786 |
| 1500-1999 | 37.3502 | 13 | 0.0004 | 13.7907 | 2 | 0.001 |
| 2000+ | 11.7515 | 6 | 0.0677 | 1.076 | 2 | 0.5839 |

**Appendix Table 6. Q values for *p*CO_2_ ranges in the Ca**^2+^ **by biogeography analysis**

| Calcium: Biogeography | | | | | | |
| --- | --- | --- | --- | --- | --- | --- |
| *p*CO_2_ range | Q_e_ | | | Q_m_ | | |
|  | Q_e_ | df | p-value | Q_m_ | df | p-value |
| Overall | 180.8193 | 48 | < .0001 | 10.8135 | 3 | 0.0128 |
| 500-999 | 29.4633 | 13 | 0.0056 | 0.3829 | 3 | 0.9437 |
| 1000-1499 | 53.827 | 9 | < .0001 | 3.0976 | 3 | 0.3768 |
| 1500-1999 | 54.7494 | 13 | < .0001 | 8.789 | 2 | 0.0123 |
| 2000+ | 11.7515 | 6 | 0.0677 | 1.076 | 2 | 0.5839 |

**Appendix Table 7. Q values for *p*CO_2_ ranges in the Ca**^2+^ **by life history stage analysis**

| Calcium: Life history stage | | | | | | |
| --- | --- | --- | --- | --- | --- | --- |
| *p*CO_2_ range | Q_e_ | | | Q_m_ | | |
|  | Q_e_ | df | p-value | Q_m_ | df | p-value |
| Overall | 157.2134 | 48 | < .0001 | 12.7641 | 3 | 0.0052 |
| 500-999 | 29.6325 | 14 | 0.0086 | 1.4936 | 2 | 0.4739 |
| 1000-1499 | 38.6745 | 9 | < .0001 | 7.2469 | 3 | 0.0644 |
| 1500-1999 | 46.975 | 13 | < .0001 | 10.0344 | 2 | 0.0066 |
| 2000+ | 3.3518 | 6 | 0.7636 | 9.3566 | 2 | 0.0093 |

**Appendix Table 8. Q values for *p*CO_2_ ranges in the Ca**^2+^ **by anatomical region analysis**

| Calcium: Anatomical Region | | | | | | |
| --- | --- | --- | --- | --- | --- | --- |
| pCO_2_ range | Q_e_ | | | Q_m_ | | |
|  | Q_e_ | df | p-value | Q_m_ | df | p-value |
| Overall | 179.2602 | 49 | < .0001 | 5.51232 | 2 | 0.0772 |
| 500-999 | 29.6581 | 14 | 0.0085 | 0.3814 | 2 | 0.8264 |
| 1000-1499 | 73.405 | 10 | < .0001 | 0.5005 | 2 | 0.7786 |
| 1500-1999 | 37.3502 | 13 | 0.0004 | 13.7907 | 2 | 0.001 |
| 2000+ | 11.7515 | 6 | 0.0677 | 1.076 | 2 | 0.5839 |

**Appendix Table 9. Q values for *p*CO_2_ ranges in the Mg**^2+^ **analysis**

| Mg model results | | | | |
| --- | --- | --- | --- | --- |
| *p*CO_2_ range (µatm) | Q | df | p-value | Model direction |
| 500-999 | 36.8728 | 13 | 0.0004 | No effect |
| 1000-1499 | 17.0004 | 8 | 0.0301 | No effect |
| 1500-1999 | 30.2356 | 13 | 0.0044 | Negative |
| 2000+ | 24.1281 | 7 | 0.0011 | No effect |

**Appendix Table 10. Q values for *p*CO_2_ ranges in the Mg**^2+^ **by taxonomic order analysis**

| Magnesium: Taxonomic Order | | | | | | |
| --- | --- | --- | --- | --- | --- | --- |
| pCO_2_ range | Q_e_ | | | Q_m_ | | |
|  | Q_e_ | df | p-value | Q_m_ | df | p-value |
| Overall | 113.5631 | 43 | < .0001 | 3.3422 | 2 | 0.188 |
| 500-999 | 29.5282 | 12 | 0.0033 | 0.6541 | 2 | 0.7211 |
| 1000-1499 | 16.5146 | 7 | 0.0208 | 0.232 | 2 | 0.8905 |
| 1500-1999 | 27.27 | 12 | 0.0071 | 6.5317 | 2 | 0.0382 |
| 2000+ | 19.0532 | 6 | 0.0041 | 4.8407 | 2 | 0.0889 |

**Appendix Table 11. Q values for *p*CO_2_ ranges in the Mg**^2+^ **by biogeography analysis**

| Magnesium: Biogeography | | | | | | |
| --- | --- | --- | --- | --- | --- | --- |
| pCO_2_ range | Q_e_ | | | Q_m_ | | |
|  | Q_e_ | df | p-value | Q_m_ | df | p-value |
| Overall | 126.4363 | 42 | < .0001 | 4.2423 | 3 | 0.2365 |
| 500-999 | 29.4742 | 11 | 0.0019 | 0.5226 | 3 | 0.9139 |
| 1000-1499 | 14.1333 | 6 | 0.0282 | 1.805 | 3 | 0.6138 |
| 1500-1999 | 13.1996 | 12 | 0.3547 | 25.4797 | 2 | < .0001 |
| 2000+ | 23.8353 | 6 | 0.0006 | 1.7179 | 2 | 0.4236 |

**Appendix Table 12. Q values for *p*CO_2_ ranges in the Mg**^2+^ **by life history stage analysis**

| Magnesium: Life History Stage | | | | | | |
| --- | --- | --- | --- | --- | --- | --- |
| pCO_2_ range | Q_e_ | | | Q_m_ | | |
|  | Q_e_ | df | p-value | Q_m_ | df | p-value |
| Overall | 132.7384 | 42 | < .0001 | 0.9099 | 3 | 0.823 |
| 500-999 | 36.0305 | 12 | 0.0003 | 0.6075 | 2 | 0.7381 |
| 1000-1499 | 16.3291 | 6 | 0.0121 | 0.4973 | 3 | 0.9195 |
| 1500-1999 | 27.27 | 12 | 0.0071 | 6.5317 | 2 | 0.0382 |
| 2000+ | 23.8353 | 6 | 0.0006 | 1.7179 | 2 | 0.4236 |

**Appendix Table 13. Q values for *p*CO_2_ ranges in the Mg**^2+^ **by anatomical region analysis**

| Magnesium: Anatomical Region | | | | | | |
| --- | --- | --- | --- | --- | --- | --- |
| pCO2 range | Qe | | | Qm | | |
|  | Qe | df | p-value | Qm | df | p-value |
| Overall | 179.2602 | 49 | < .0001 | 5.51232 | 2 | 0.0772 |
| 500-999 | 29.6581 | 14 | 0.0085 | 0.3814 | 2 | 0.8264 |
| 1000-1499 | 73.405 | 10 | < .0001 | 0.5005 | 2 | 0.7786 |
| 1500-1999 | 37.3502 | 13 | 0.0004 | 13.7907 | 2 | 0.001 |
| 2000+ | 11.7515 | 6 | 0.0677 | 1.076 | 2 | 0.4236 |

**Appendix Table 14. Q values for *p*CO_2_ ranges in the biomechanics analysis**

| Biomechanical properties | | | | |
| --- | --- | --- | --- | --- |
| *p*CO_2_ range (µatm) | Q | df | p-value | Model direction |
| 500-999 | 26.5254 | 11 | 0.0054 | Negative |
| 1000-1499 | NA | NA | NA | NA |
| 1500-1999 | 25.6321 | 5 | 0.0001 | Negative |
| 2000+ | 0.00 | 0 | **1.0000** | No effect |

**Appendix Table 15. Q values for *p*CO_2_ ranges in the cuticle thickness analysis**

| Total cuticle thickness | | | | |
| --- | --- | --- | --- | --- |
| *p*CO_2_ range (µatm) | Q | df | p-value | Model direction |
| 500-999 | 9.2244 | 9 | 0.4168 | No effect |
| 1000-1499 | 0.00 | 0 | **1.0000** | No effect |
| 1500-1999 | 8.9427 | 5 | 0.1114 | No effect |
| 2000+ | 0.00 | 0 | **1.0000** | No effect |
